# Structural Characterization and Anticoagulant Potential of *Colochirus quadrangularis* Fucosylated Glycosaminoglycan 5−12 Oligomers with Unusual Branches

**DOI:** 10.3390/md23020064

**Published:** 2025-02-01

**Authors:** Xuedong Zhang, Guangwei Yan, Xinming Liu, Jiewen Fu, Xiang Shi, Pei Cao, Yuqian Sun, Shengping Zhong, Jiale Nong, Peiqi Jiang, Yonghong Liu, Baoshun Zhang, Qingxia Yuan, Longyan Zhao

**Affiliations:** 1 Guangxi Key Laboratory of Marine Drugs, Institute of Marine Drugs, Guangxi University of Chinese Medicine, Nanning 530200, China; z154535199@163.com (X.Z.); liuxm@gxtcmu.edu.cn (X.L.); weiwei1999777@163.com (J.F.); sx20200807@163.com (X.S.); caopeicib@hotmail.com (P.C.); shpzhong@foxmail.com (S.Z.); njll18376917013@163.com (J.N.); jiangpeiqi304@foxmail.com (P.J.); yonghongliu@scsio.ac.cn (Y.L.); 2 College of Pharmaceutical Sciences, Southwest University, Chongqing 400716, China; ygw19981208@163.com; 3 Instrumentation and Service Center for Molecular Sciences, Westlake University, Hangzhou 310024, China; sunyuqian@westlake.edu.cn

**Keywords:** sea cucumber, fucosylated glycosaminoglycan, oligosaccharides, chemical structure, anticoagulant activity

## Abstract

The depolymerized products and oligosaccharide fractions from sea cucumber fucosylated glycosaminoglycans (FGs) are promising anticoagulant candidates, and more novel FG-derived oligosaccharides from low-priced sea cucumbers are expected to be obtained. This study isolated 5−12 oligomers (OF1−OF3) with unusual branches from β-eliminative depolymerized products of *Colochirus quadrangularis* FG (CqFG). Detailed NMR analyses showed that OF1−OF3 consisted of a chondroitin 4,6-sulfates backbone and some sulfated fucosyl branches (FucS), including monosaccharides (α-l-Fuc_2S4S_, α-l-Fuc_3S_, α-l-Fuc_4S_, α-l-Fuc_2S3S4S_, and α-l-Fuc_2S_) and a disaccharide D-Gal_3S4S_-α1,3-l-Fuc_2S4S_ with the ratio of ~36:35:10:7:3:9, attached to the C-3 position of β-d-GlcA or its derivatives, such as α-l-Δ4,5GlcA and β-d-GlcA-ol. Unusually, α-l-Fuc_3S_ was the main FucS branch; no α-l-Fuc_3S4S_ branch was found, and α-l-Fuc_2S3S4S_ and α-l-Fuc_2S_ branches were also found in OF1–OF3. The OF2 and OF3 could strongly inhibit the intrinsic and common coagulation pathways. Intrinsic FXase is a target of OF2 and OF3 inhibiting the intrinsic coagulation pathways, and the unusual side chains may increase the intrinsic FXase inhibitory activity. OF2 and OF3 showed negligible bleeding risk, and less bleeding than heparin (HP), low-molecular-weight heparins (LMWHs), and CqFG. These findings support novel FG oligosaccharides with some unusual branches from low-priced sea cucumbers to be prepared as safer anticoagulants.

## 1. Introduction

Thrombotic cardiovascular disease is the major cause of death worldwide, and 18 million people die from this disease each year, accounting for 30 percent of global deaths. Heparin is an essential drug listed by the WHO and has made outstanding contributions to the clinical treatment of thrombotic diseases. However, heparin drugs including low-molecular-weight heparin (LMWH) are mostly derived from mammalian viscera, which faces the safety problem of raw materials potentially caused by prions, swine fever, and other pyrogens [1,2]. They also have serious fatal safety problems, such as bleeding tendency and thrombocytopenia risk [3]. Therefore, in recent decades, researchers have continued looking for safer anticoagulants from marine organisms, such as sea cucumbers and algae [4,5].

Fucosylated glycosaminoglycans (FGs) from sea cucumbers have strong anticoagulant activity and have attracted great attention as a lead compound of new anticoagulant drugs for over 40 years [6,7]. In the last decade, great progress has been made in low-molecular-weight FG (LFG) preparation by glycosidic bond-selective depolymerization methods, such as partial N-deacetylation–deaminative cleavage and β-eliminative depolymerization, and in the purification of oligosaccharide fragments [5,8,9,10]. The LFG-53/YB209 containing sulfated fucose (Fuc) monosaccharide branches developed as a new chemical class 1.1 drug was approved for clinical study by the Food and Drug Administration (FDA) in the USA and National Medical Products Administration (NMPA) in China in 2021 and 2022, respectively [6,11]. Structural variations and the complexity of FGs are species-specific. Generally, FGs from most reported sea cucumber species including LFG-53 contain only different proportions of Fuc_3S4S_, Fuc_2S4S_, and Fuc_4S_ monosaccharide branches [6]. In recent years, the long-standing question about disaccharide-branched fucosylated glycosaminoglycans (FGs) in sea cucumbers has been gradually clarified by structural elucidation of the purified oligosaccharides containing several unusual disaccharide branches, including D-GalNAc_4S(6S)_-α1,2-l-Fuc_3S_, D-GalNAc-α1,2-l-Fuc_3S4S_, D-Gal_4S(6S)_-α1,2-l-Fuc_3S_, D-GalNAc_4S(6S)_-α1,2-l-Fuc_3S(4S)_, and α-d-GalNAc-1,2-α-l-Fuc_3S4S_ found in FGs from the sea cucumber species, such as *Holothuria nobilis*, *Acaudina molpadioides*, *Thelenota ananas*, *Ludwigothurea grisea*, *Phyllophorella kohkutiensis*, *Stichopus monotuberculatus*, and *Holothuria mexicana* [11,12,13,14,15,16,17]. These purified oligosaccharides act only on intrinsic coagulation pathways, and the proportions of sulfated monosaccharide and disaccharide branches may influence their anticoagulant activity [5,6]. According to previous studies, most research on FGs focused on high-priced edible sea cucumbers [6]. It is significant to continue preparing novel purified oligosaccharides from some low-priced inedible sea cucumber species to develop new safe anticoagulant drugs.

*Colochirus quadrangularis* belongs to Holothuroidea Cucumariidae Colochirus and is mainly distributed in the Indo-Pacific region, especially in the Weizhou Island of Beibu Gulf of Guangxi, China [18]. It is rich in resources and low-priced but has the characteristics of low edibility. Moreover, no information is available on the polysaccharides and oligosaccharides from this sea cucumber species. Therefore, in this study, 5−12 oligomers from *C. quadrangularis* FG (CqFG) were obtained by β-eliminative depolymerization and column chromatography. Their structures were elucidated, the characteristics of anticoagulant activity were determined, and their bleeding risks were evaluated, which may provide valuable reference information for the research and development of new anticoagulant drugs.

## 2. Results and Discussion

### 2.1. Preparation and Physicochemical Properties of CqFG, dCqFG, and Oligosaccharide Fractions

**Figure 1:**
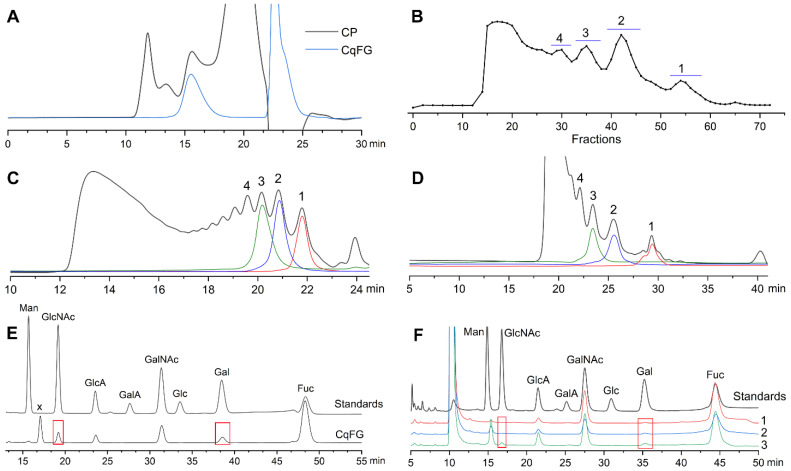
High-performance liquid chromatography (HPLC) analyses of CqFG, dCqFG, and oligosaccharide fractions. HPLC profiles of crude polysaccharides (CP) and CqFG (**A**), elution curve of dCqFG by Bio-Gel P10 column (**B**), profiles of dCqFG and OF1–OF3 determined by TSKgel G2000WXL column (**C**) and Superdex™ 30 Increase 10/300 GL column (**D**), and chromatograms of PMP derivatives of CqFG (**E**) and OF1–OF3 (**F**).

After extraction and purification, the yield of CqFG was 1.07% based on the dried *C. quadrangularis* powder. The profile of CqFG showed a single chromatographic peak. The molecular weight (*M*_w_) was calculated to be 77.7 kDa with a polydispersity index of 1.7 (Figure 1A). According to its ^1^H NMR spectrum in Figure S1, the integration ratio of signals from protons in the acetyl group (3H) in N-acetyl-d-galactosamine (D-GalNAc) (~2.07 ppm) and methyl group (3H) in Fuc (~1.3 ppm) was 1:3.39, suggesting that fucan sulfate (FS) may exist in CqFG. CqFG was depolymerized by the β-eliminative depolymerization method. The glycosidic bonds of GalNAc-β1,4-GlcA in the CqFG could be selectively cleaved, leading to the formation of dCqFG with a Δ4,5GlcA residue at the new non-reducing end of resulting oligosaccharide fragments [10,12]. The elution curves determined by the Bio-Gel P10, TSKgel G2000WXL, and Superdex™ 30 Increase 10/300 GL columns indicated that dCqFG contained some relatively regular oligosaccharide fragments with different *M*_w_ (Figure 1B–D). Based on their molecular size, the oligosaccharide fractions (OF) 1–4 in the dCqFG were isolated by the Bio-Gel P6 and P10 columns, and OF1–OF3 were chosen for further investigation. OF1–OF3 all showed a single peak on the TSKgel G2000WXL column (Figure 1C), and OF1 displayed three oligosaccharide peaks on the Superdex™ 30 Increase 10/300 GL column for its higher resolution for analyzing low-molecular-weight oligosaccharides or polypeptides (Figure 1D). Based on the retention times of various oligosaccharides isolated from *Stichopus Variegatus* and *S. monotuberculatus* in our previous study [9,15], OF1–OF3 mainly contained pentasaccharides, octasaccharides, and hendecasaccharides, respectively. The high-molecular-weight fraction (*M*_w_ > 10 kDa) with a retention time between approximately 12 and 17 min in Figure 1C was further confirmed to be an FS by the ^1^H NMR spectrum displaying strong -CH_3_ signals in the region of 1.2–1.5 ppm and almost no -CH_3_ signals of acetyl groups at ~2.06 ppm (Figure S2). This result further confirmed that the β-eliminative depolymerization cannot depolymerize the FS. The FSs with various structures are widely present in all species of sea cucumbers, and some of them have similar molecular weight distribution and charge numbers with FG [19], resulting in the FS contained in FG that cannot be isolated and removed by anion exchange chromatography and gel permeation chromatography (GPC). Therefore, in some studies, sulfated Fuc (FucS) side chains in the form of a disaccharide, trisaccharide, and even nonasaccharide linked to the backbone of FGs were proposed, probably due to being misled by the FS [20,21,22].

As shown in Figure 1E, the monosaccharide composition analysis indicated that CqFG comprised D-glucuronic acid (GlcA), GalNAc, Fuc, N-acetyl-d-glucosamine (GlcNAc), and D-galactose (Gal). According to previous studies, FGs from most reported sea cucumbers, such as *S. variegatus*, *Apostichopus japonicus*, and *Holothuria fuscopunctata* [6,9,23,24], are only composed of GlcA, GalNAc, and Fuc, but do not contain GlcNAc or Gal. In 2021, it was reported that FG from *T. ananas* contained Gal [12]. However, GlcNAc is unusual and has not been found in FG. A peak (marked x) with a retention time between D-mannose (Man) and GlcNAc was a 3-Methyl-1-phenyl-2-pyrazolin-5-one (PMP)-labeled acidolysis-resistant disaccharide with a mass-to-charge peak of [M-H]^−^ (i.e., 684.2438, calculated m/z 684.2595) (Figure S3). OF3 contained Gal and GlcNAc. However, GlcNAc was not observed in OF1 and OF2, which may indicate their low proportion in natural CqFG (Figure 1F).

### 2.2. Structural Analysis of Oligosaccharide Fractions

**Figure 2:**
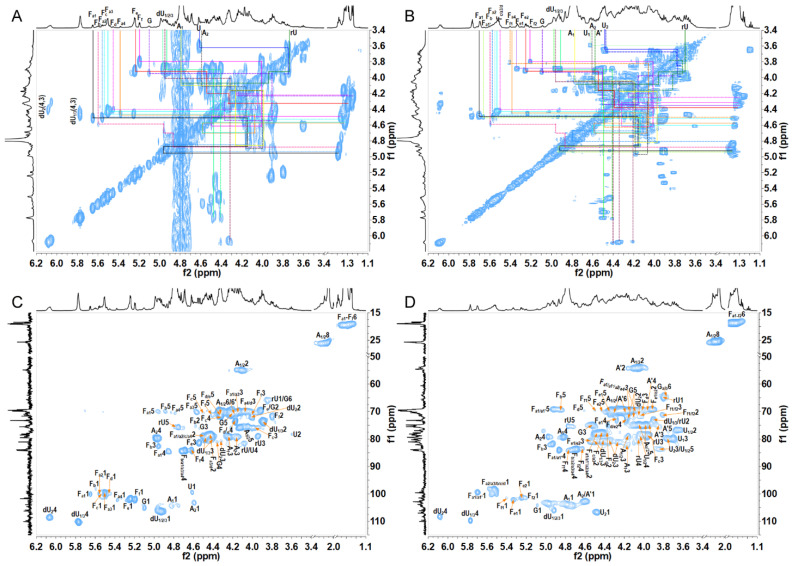
^1^H-^1^H COSY and ^1^H-^13^C HSQC spectra of fractions 1 (OF1) (**A**,**C**) and (OF3) (**B**,**D**).

**Figure 3:**
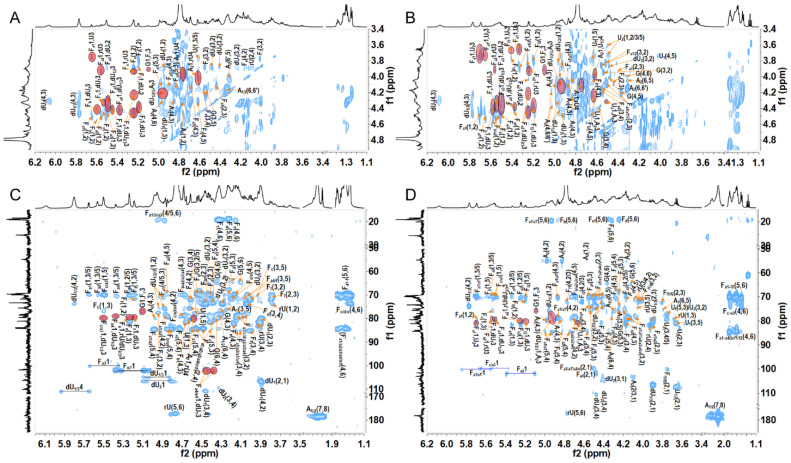
^1^H-^1^H ROESY and ^1^H-^13^C HMBC spectra of OF1 (**A**,**C**) and OF2 (**B**,**D**). The correlation signals in the red ellipse indicate the connection positions between two sugar residues.

All the ^1^H and ^13^C signals in the ^1^H-^1^H COSY, TOCSY, ROESY, ^1^H-^13^C HSQC, HSQC-TOCSY, and HMBC spectra of OF1–OF3 (Figure 2, Figure 3 and Figures S4–S12) can be assigned based on the data from other common sea cucumber species as shown in previous studies [9,10,12]. The complete assignments of each sugar residue’s proton and carbon resonances are shown in Table 1 and Tables S1 and S2. The sugar residues with anomeric proton signal at approximately 5.20–5.70 ppm and anomeric carbon signal at approximately 99–102 ppm were assigned to different sulfation types of α-l-Fuc residues (F_a1_–F_f2_). F_a1-a4_ could be deduced as α-l-Fuc_2S4S_ due to their characteristic downfield chemical shifts in H-2 and H-4 and different chemical environments [9]. Similarly, F_b_, F_c_, F_d_, F_e1/e2_, and F_f1/f2_ could be deduced as α-l-Fuc_2S3S4S_, α-l-Fuc_2S4S_, α-l-Fuc_2S_, α-l-Fuc_3S_, and α-l-Fuc_4S_, respectively [16,17]. The signals at approximately (4.65, 105.2), (4.49, 107.3), (4.93–4.96, 104.4–106.5), and (3.73/3.84, 65.7) ppm shown in the HSQC spectra (Figure 2C,D and Figure S10) were assigned to the H-1/C-1 of β-d-GlcA, Δ^4,5^ unsaturated glucuronic acids (α-l-Δ^4,5^GlcA) derived from GlcA during the β-eliminative depolymerization, and β-d-GlcA-ol (rU) produced by the peeling reaction during the β-eliminative depolymerization [10]. The signals at (4.59, 103.2) and (4.79, 104.5) ppm were from the anomeric signal of β-GalNAc_4S6S_ (A). The location of the sulfated group at both C-4 and C-6 positions on the β-GalNAc residues could be identified from their obvious downfield shifts in protons and carbons [13]. The sugar residue G shown in Figure 2 and Figure S10 with an anomeric proton signal at approximately 5.09 ppm and a relatively larger chemical shift (105 ppm) than FucS residues could be deduced as α-Gal residue [12]. Its values of δ_H-3_ at 4.54 ppm and δ_H-4_ at 4.36 ppm were shifted downfield compared with those of non-sulfated Gal residues, indicating that the residue G was 3-*O*-sulfated and 4-*O*-sulfated. The residue A’ from OF3 with δ_H1/C1–H6/C6_ at 4.58/103.14, 4.15/54.37, 3.84/75.41, 3.95/70.88, 3.75/77.40, and 4.17/4.26/69.73 ppm could be deduced as β-d-GlcNAc_6S_ residue.

**Figure 4:**
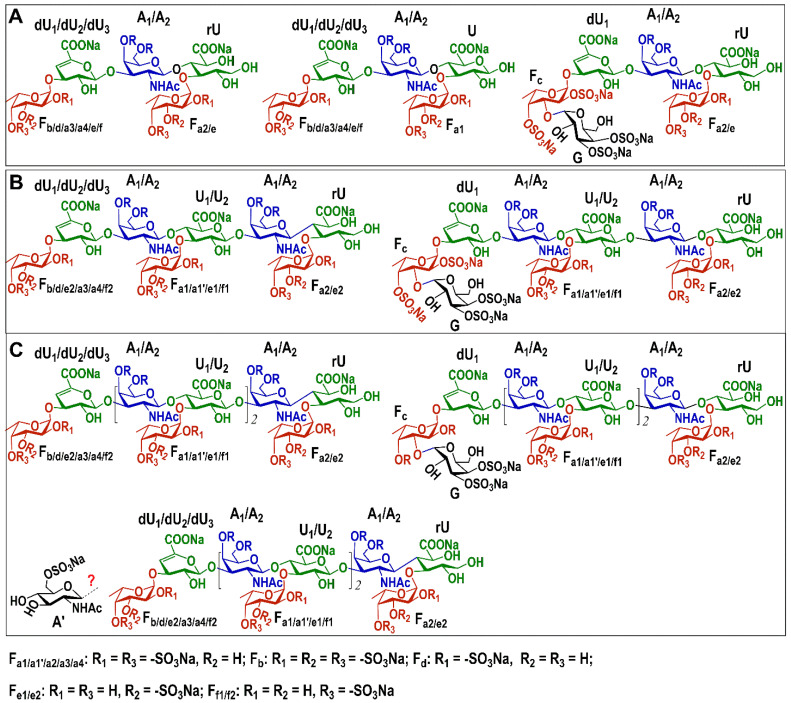
Proposed structures of OF1 (**A**), OF2 (**B**), and OF3 (**C**). The red mark “?” represents an uncertain connection pattern.

The order of various sugar residues in OF1–OF3 could be deduced according to the correlation peaks in their ^1^H-^1^H ROESY and ^1^H-^13^C HMBC spectra (Figure 3 and Figure S12) and shown in Table 1 and Tables S1 and S2. H/C-1 of residues dU_1/2/3_ and U_1/2_ showed cross-peaks to H/C-3 of residue A_1/2_; H/C-1 of residues A_1/2_ showed cross-peaks to H/C-4 of residue U_1/2_ or rU; and H/C-1 of all FucS residues F_a1_–F_f2_ showed cross-peaks to H/C-3 of residue dU_1/2/3_, U_1/2_, or rU. The signals at 5.09/3.92 ppm in the ROESY spectra and 5.09/75.77 ppm in the HMBC spectra of OF1–OF3 could confirm that C-1 of G was linked to the C-3 position of Fc. In the OF3, the cross-peak 4.58/83.82 ppm in the HMBC spectrum suggested that residue A′ may attach to the C-3 position of U_2_ (Figure S12). The β-d-GlcNAc_6S_ in OF3 may exist as a novel branch, but its content is too low, and more convincing evidence should be provided in the future. Hence, the structures of OF1–OF3 were deduced as shown in Figure 4.

According to the results of previous studies on the structure of FGs from various sea cucumbers, all the FGs including their derived oligosaccharides have a chondroitin sulfate (CS)-like backbone formed alternately by -4)-β-d-GlcA-(1- and -3)-β-d-GalNAc-(1- [6,7]. Various sulfation types of Fuc side chains are linked to the C-3 position of β-d-GlcA or the C-4/6 position of β-d-GalNAc in the backbone. In this study, OF1–OF3 were composed of a chondroitin 4,6-sulfates (CS-E) backbone. The FucS monosaccharide (α-l-Fuc_2S4S_, α-l-Fuc_3S_, α-l-Fuc_2S3S4S_, α-l-Fuc_2S_, and α-l-Fuc_4S_) and disaccharide D-Gal_3S4S_-α1,3-l-Fuc_2S4S_ side chains were linked to the C-3 position of β-d-GlcA or its derivatives, such as α-l-Δ^4,5^GlcA and β-d-GlcA-ol. Hence, OF1 was a mixture of penta- and hexa-saccharides. OF2 was a mixture of octa- and nona-saccharides, and OF3 was a mixture of hendeca- and dodeca-saccharides, which added one or two trisaccharides (FucS-α1,3-GlcA-β1,3-GalNAc) in the middle of the main chain of OF1. The integration ratios of signals from protons in the acetyl group (3H) in GalNAc (~2.06 ppm) and the methyl group (3H) in Fuc (~1.3 ppm) obtained from ^1^H NMR spectra of OF1–OF3 (Figures S4, S7, and S11) were 1:2.0, 1:1.4, and 1:1.2, respectively, further confirmed these inferences. According to the anomeric proton peak area ratios of FucS residues in the ^1^H NMR spectrum of OF1 (Figure S4), α-l-Fuc_2S4S_, α-l-Fuc_3S_, α-l-Fuc_4S_, α-l-Fuc_2S3S4S_, and α-l-Fuc_2S_ as a monosaccharide side chain accounted for 35.8%, 34.8%, 10.3%, 7.4%, and 2.9%, respectively. The disaccharide side chain (D-Gal_3S4S_-α1,3-l-Fuc_2S4S_) accounted for 8.8%. α-l-Fuc_2S4S_, α-l-Fuc_3S_, α-l-Fuc_4S_, α-l-Fuc_2S3S4S_, α-l-Fuc_2S_, and the disaccharide side chain in OF2 accounted for 35.8%, 32.3%, 10.7%, 9.3%, 2.8%, and 9.0%, respectively (Figure S7). OF3 had similar percentage compositions of these side chains as OF1 and OF2, accounting for 35.9%, 30.1%, 9.4%, 6.8%, 2.3%, and 8.9%, respectively (Figure S11).

In the last ten years, many kinds of oligosaccharides were prepared from some sea cucumbers, such as *S. variegatus*, *Bohadschia argus*, *Actinopyga miliaris*, *Holothuria albiventer*, *Holothuria coluber*, *Pearsonothuria graeffei*, *Isostichopus badionotus*, *A. molpadioides*, *S. monotuberculatus*, *T. ananas*, *L. grisea*, and *P. kohkutiensis*, using the glycosidic bond-selective depolymerization methods, including the deacetylation–deaminative cleavage and β-eliminative depolymerization [6,9,13,15,16,17]. These oligosaccharides have some common structural features, such as a CS-E-like backbone, FucS-containing monosaccharide (or disaccharide) side chains linked to C-3 position of β-d-GlcA in the backbone, and each β-d-GlcA linked by FucS at the C-3 position. In our present study, OF1–OF3 derived from C. quadrangularis also had the same structural characteristics. In previous studies, some unusual structural features, such as CS-A and CS-C units existed in the backbone, FucS side chains located at the C-4/C-6 position of GalNAc, and C-2/C-3 positions of GlcA linked by sulfate group, were proposed by analyzing the native FG or its depolymerized product from some cucumber species, including *H. Mexicana*, *Cucumaria japonica*, and *Hemioedema spectabilis* [25,26,27]. However, as a complex biomacromolecule, FG and its depolymerized product have overlapping NMR spectra with broad signals, thus hindering the elucidation of fine structures and resulting in these controversial proposed structural characteristics. An in-depth and detailed study on the structural analysis of FG from *H. Mexicana* provided more convincing evidence by analyzing the precise structures of purified oligosaccharides derived from this FG and therefore contradicted the structural characteristics proposed by other groups [11]. Studies using oligosaccharide fractions with clear structural features may allow for more definitive conclusions.

In this study, we confirmed that OF1–OF3 had some unusual structural characteristics of side chains, significantly different from those of previously reported sea cucumbers [6]. OF1–OF3 contained a large amount of α-l-Fuc_3S_ side chains (~35%), close to the content of α-l-Fuc_2S4S_ side chains. According to previous studies, FGs from most sea cucumbers do not contain α-l-Fuc_3S_ side chains [6]. Although some studies on NMR analyses of the intact FG and partial hydrolysate mixture proposed that FGs from *T. ananas*, *L. grisea*, and *H. nobilis* contained a high content of α-l-Fuc_3S_ monosaccharide side chains [28,29], their structures were revised in recent years by oligosaccharide isolation and clearer structural analysis, indicating that the content of α-l-Fuc_3S_ side chains is very low [12,13,17]. So far, no purified oligosaccharide fractions (dp > 5) with a high content of α-l-Fuc_3S_ have been obtained. Unexpectedly, OF1–OF3 do not contain the Fuc_3S4S_ side chain common in FG-derived oligosaccharides from other sea cucumbers. Moreover, unusual α-l-Fuc_2S3S4S_ monosaccharide and D-Gal_3S4S_-α1,3-l-Fuc_2S4S_ disaccharide side chains have been found in FG-derived oligosaccharide fractions for the first time. To date, preparing FG-derived oligosaccharides containing unusual branches by chemical synthesis for the structure–activity relationship study still cannot be achieved. Therefore, these oligosaccharide fractions OF1–OF3 obtained in this study may play an important role in the research and development of anticoagulant drugs.

### 2.3. Anticoagulant Activity of OF1–OF3 and Their Effects on Human Intrinsic Factor Tenase Complex (FXase)

Activated partial thromboplastin time (APTT), thrombin time (TT), and prothrombin time (PT) of plasma clotting assays were used to examine the anticoagulant activity of OF1–OF3. As shown in Table 2 and Figure S13, HP and CqFG showed strong APTT-, TT-, and PT-prolonging activities, indicating that they could inhibit blood clotting through the intrinsic, common, and extrinsic pathways of the coagulation cascade. It is reported that anticoagulants that inhibit coagulation factors in the extrinsic coagulation pathway may affect physiological hemostasis and therefore have a serious bleeding risk [5]. CqFG showed higher PT-prolonging activity than LMWHs and FGs from some sea cucumbers, such as *S. variegatus* and *H. fuscopunctata* [9,30]. OF2 and OF3 displayed strong APTT- and TT-prolonging activities; in particular, their APTT-prolonging activities were stronger than or close to that of LMWHs. According to our previous studies, the purified oligosaccharide fractions with dp up to 18 mainly containing L-Fuc_2S4S_ or L-Fuc_3S4S_ have no TT-prolonging activity within the concentration range of 0–128 μg/mL [9,31]. These results suggested that the FG-derived oligosaccharides containing higher contents of α-l-Fuc_3S_ and other unusual side chains have stronger anticoagulant activity and exhibit stronger effects on the common coagulation pathway. The unusual side chains, including α-l-Fuc_2S3S4S_, β-d-GlcNAc_6S_, and D-Gal_3S4S_-α1,3-l-Fuc_2S4S_, may contribute to the anticoagulant activities. The results also indicated that the anticoagulant activity of oligosaccharide fractions from CqFG increased with the increase in *M*_w_. The lower *M*_w_ of oligosaccharide fraction OF1 did not affect TT at concentrations as high as 128 μg/mL but still had anticoagulant activity with a concentration of 34.95 μg/mL required to double the APTT.

### 2.4. Bleeding Risk of OF3

**Figure 5:**
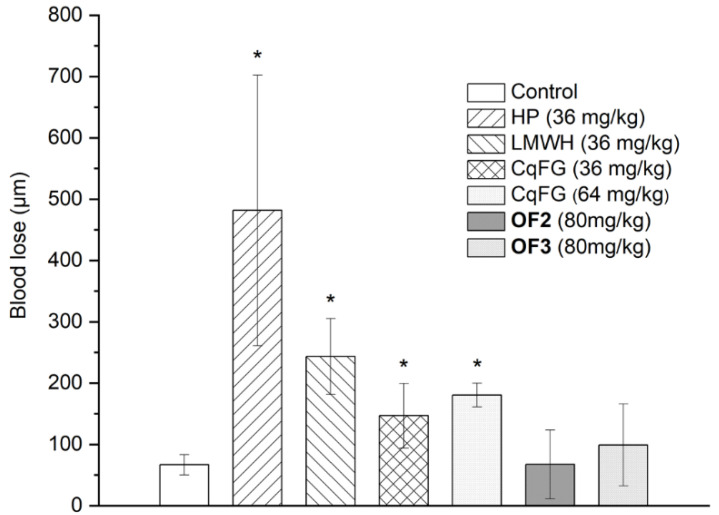
Effects of OF2 and OF3 on blood loss in mice (*n* = 4, * *p* < 0.05 vs. control).

The bleeding risk of CqFG, OF2, and OF3 in vivo was determined for this side effect and is the top concern for developing a new anticoagulant drug. As shown in Figure 5, HP and LMWH significantly increased blood loss of the mice (*p* < 0.05) at a dose of 36 mg/kg more than 10-fold the dose for thrombus inhibition (>80% inhibition rate), compared with those of the mice in the normal control group. CqFG at the same dose as LMWH (36 mg/kg) or at a higher dose of 64 mg/kg significantly increased the bleeding volumes of mice compared with the control group (*p* > 0.05), which is consistent with that of FG from *H. fuscopunctata* [30]. OF2 and OF3 at a dose as high as 80 mg/kg did not significantly increase the bleeding volumes of mice compared with the control group (*p* > 0.05). These results indicated that OF2 and OF3 have lower bleeding risk than HP, LMWH, and CqFG, and may be safer, promising anticoagulants that do not cause serious bleeding.

## 3. Materials and Methods

### 3.1. Materials and Reagents

The fresh *C. quadrangularis* were fished from the sea near Weizhou Island, Beihai City, Guangxi Zhuang Autonomous Region, China. The sea cucumber species was identified by Professor Xinming Liu and Jing Wen from Guangxi University of Chinese Medicine and Shaoguan University, respectively. Amberlite FPA98Cl was obtained from Rohm and Haas Company (Philadelphia, PA, USA). Sepharose CL-6B, Sephadex G-25 gel columns, and Superdex™ 30 Increase 10/300 GL were obtained from GE Healthcare Life Sicences (Marlborough, USA). Bio-gel P-series gel columns including P-2, P-6, and P-10 were purchased from Bio-Rad Laboratories (Hercules, CA, USA). D-GalNAc, L-Fuc, and D-Man were obtained from Aladdin Chemical Reagent Co., Ltd. (Shanghai, China). Standard SEC-Pullulan was obtained from Sepax Technologies Inc. (Newark, DE, USA). D-glucose, D-GlcA, D-Gal, D-galacturonic acid, D-GlcNAc, deuterium oxide (D_2_O) with 99.9% atom D, PMP, and HP were purchased from Sigma-Aldrich (China). The CaCl_2_ solution (0.05 M), coagulation control plasma, and APTT, TT, and PT assay kits were obtained from TICO GmbH (Germany). Tris-HCl (>99.5%) was purchased from Amresco (Solon, OH, USA). Enoxaparin (LMWH, *M*_w_ ~ 4500 Da, 0.4 mL × 4000 AXaIU) was from Sanofi-Aventis (France). Biophen FVIII: C kit was purchased from Hyphen Biomed (Paris, France). Human FVIII was obtained from Bayer Healthcare LLC (Leverkusen, Germany). Kunming mice (male, body weight 18 ± 2 g) were purchased from Hunan SJA Laboratory Animal CO., LTD (Changsha, China). All other chemicals obtained commercially were of analytical grade.

### 3.2. Extraction, Isolation, and Physicochemical Analysis of CqFG

The extraction and isolation of CqFG were carried out, as described in our previous study [8,15]. The intestinal tract of *C. quadrangularis* was removed, and the body wall (5.6 kg) was chopped, dried at 60 °C, crushed, and extracted by 0.2% papain solution at 55 °C for 16 h and 0.5 M NaOH solution for 2 h. After centrifugation (4700 rpm × 15 min), the supernatant was adjusted to pH 3 and stood at 4 °C 6 h to remove protein. After centrifugation, the supernatant was adjusted to pH 7.0, and ethanol was added to a final concentration of 75% (v/v). The crude polysaccharide was obtained by centrifugation and lyophilization. After purification by strong anion exchange chromatography with FPA98Cl resin, the 2.0 M NaCl eluate was collected, dialyzed (3.5 kDa cut-off membrane), and purified by GPC with Sepharose CL-6B (1.5 cm × 150 cm). Finally, the CqFG fractions were combined, dialyzed, and lyophilized. 

The molecular weight distribution and monosaccharide composition of CqFG were analyzed by HPLC using a Shodex OH-pak SB-804 HQ column (8.0 mm × 300 mm) and an Eclipse Plus C18 column (5 μm, 4.6 × 250 mm), respectively. The PMP derivatization procedures and chromatographic conditions were performed as described in our previous study [4]. The sulfate content of CqFG was measured using a conductimetric titration method.

### 3.3. Preparation of dCqFG and Isolation of Oligosaccharides

The dCqFG was prepared by a β-eliminative depolymerization method as described previously [10,12,32]. Briefly, 600 mg of CqFG was dissolved in 9 mL of H_2_O, slowly added to a benzalkonium chloride solution (1.5 g in 24 mL of H_2_O), stood at 4 °C for 12 h, and centrifuged at 4000 rpm for 15 min. After centrifugation (4700 rpm × 15 min), the precipitation was washed with H_2_O and dried at 40 °C in a vacuum oven to obtain a CqFG quaternary ammonium salt (1.6 g). The sample was dissolved in 7.2 mL of DMF, 340 μL of benzyl chloride was added, and the mixture was reacted at 35 °C for 24 h. The reaction solution was cooled to 25 °C, 2.89 mL of 0.08 M EtONa/EtOH solution was added to the final concentration of 0.02 M, and the mixture was reacted for 30 min. An equal volume of saturated NaCl solution and a final concentration of 80% (V/V) alcohol were added. After centrifugation, the residue was dissolved in H_2_O, the NaOH solution and sodium borohydride were successively added to a final concentration of 0.1 M, and the mixture was reacted at 25 °C for 30 min. After neutralization, desalination, and lyophilization, dCqFG was obtained and analyzed by HPLC using TSKgel G2000WXL and SuperdexTM 30 Increase 10/300 GL columns.

The oligosaccharide fractions in the dCqFG were isolated by GPC with Bio-Gel P-6 or P-10 gel columns, as described in the literature [9]. The NaCl solution (0.2 mol/L) was used as eluent, the flow rate was 8.0 mL/h, and the fraction volume was 3 mL/tube. According to the elution curve assayed by the phenol-sulfuric acid method, each eluted peak containing oligosaccharides of the same size was pooled. After desalination and lyophilization, the 5–12 oligomer powders were obtained and analyzed by HPLC using TSKgel G2000WXL and SuperdexTM 30 Increase 10/300 GL columns.

### 3.4. Nuclear Magnetic Resonance Analysis

The NMR analyses of CqFG, dCqFG, and the purified oligosaccharide fractions were performed by a Bruker AVANCE NEO 600 M spectrometer equipped with a QCI-F cryoprobe at 298 K [33]. About 5 mg of each lyophilized sample was dissolved in 0.5 mL of D_2_O and loaded into a nuclear magnetic tube to perform the ^1^H NMR spectrum measurement. For determining the 2D NMR spectra measurement of the purified oligosaccharides, samples (10–20 mg) were dissolved in 0.5 mL of D_2_O and lyophilized. This step was repeated three times to replace exchangeable protons with D_2_O. The samples were then dissolved in 0.5 mL of D_2_O containing an internal standard 3-(trimethylsilyl) propionic-2,2,3,3-d4 acid (TSP) sodium salt for NMR analyses. The ^13^C, DEPT-135, ^1^H-^1^H COSY, TOCSY, ROESY, ^1^H-^13^C HSQC, and HMBC spectra were recorded in the indirect dimension with 1200, 512, 4, 8, 32, 4, and 64 scans, respectively.

### 3.5. Determination of Anticoagulant Activities

The anticoagulant activities of oligosaccharide fractions were evaluated by determining the APTT, TT, and PT according to the instructions of kits using standard human plasma as previously described [8,9]. Briefly, 5 μL of oligosaccharide fractions with different concentrations and standard human plasma (45 μL) were pipetted to a 37 °C preheated test tube and incubated at 37 °C for 2 min. Fifty microliters of APTT, TT, or PT reagent was added to the mixture. TT or PT was measured immediately. To determine the APTT, the mixture was incubated for 3 min, 50 μL of preheated calcium chloride (0.02 mol/L) was pipetted, and the clotting time was recorded immediately.

### 3.6. Determination of Effects of Oligosaccharide Fractions on Human Intrinsic FXase

Human intrinsic FXase inhibitory activity was determined by a chromogenic substrate method using BIOPHEN FVIII: C kit on a VICTOR Nivo multimode reader, as previously described [9,15,34]. Tris-HCl buffer solution was used as a negative control, and the activities (IC_50_) were expressed by the concentration required to inhibit half of the maximum activity.

### 3.7. Bleeding Tendency Determination of OF2 and OF3

The bleeding tendency of CqFG, OF2, and OF3 was determined using a mouse tail-bleeding model as described in the literature [9]. Kunming mice (body weight 18 ± 2 g) were housed under standard specific pathogen-free (SPF) conditions. The 64, 80, and 36 mg/kg doses for CqFG, oligosaccharide fractions (OF2 and OF3), and LMWHs were set and subcutaneously injected into the mice. After 1 h, the mouse tails were cut 5 mm from the tip and immersed in 40 mL of distilled water (37 °C) for 1.5 h. The hemoglobin in the water was determined at 540 nm, and the volume of blood loss was calculated from a standard curve at the same wavelength.

### 3.8. Statistical Analysis

The data were analyzed using the OriginPro 2021 software. One-way analysis of variance (One-Way ANOVA) followed by Duncan’s new multiple-range test was performed to determine the significance. All values were expressed as means ± SD. P values less than 0.05 (* *p* < 0.05) were considered statistically significant for the bleeding experiment. 

## 4. Conclusions

To develop new anticoagulant drugs and study the structure–activity relationship of sea cucumber FGs, such as the research on the effect of side chains on activity, requires various types of oligosaccharides with different side chains. In this study, CqFG was extracted and isolated from *C. quadrangularis*, and the oligosaccharide fractions OF1–OF3 mainly contained pentasaccharides, octasaccharides, and hendecasaccharides, respectively, which were isolated from a β-elimination depolymerized product of CqFG. Structural analysis indicated that the OF1–OF3 had a backbone of CS-E (i.e., GalNAc sulfated at both C-4 and C-6 positions). Various monosaccharide or disaccharide side chains, including α-l-Fuc_2S4S_ (~36%), α-l-Fuc_3S_ (~35%), α-l-Fuc_4S_ (~10%), α-l-Fuc_2S3S4S_ (~7%), α-l-Fuc_2S_ (~3%), and D-Gal_3S4S_-α1,3-l-Fuc_2S4S_ (~9%) linked to β-d-GlcA, α-l-Δ^4,5^GlcA, or β-d-GlcA-ol by α1,3-glycosidic bonds. The oligosaccharide fractions from CqFG containing a high content of α-l-Fuc_3S_ but no α-l-Fuc_3S4S_ were obtained for the first time. The Fuc_2S3S4S_ and D-Gal_3S4S_-α1,3-l-Fuc_2S4S_ side chains were also unusual in FGs from sea cucumbers. The anticoagulant assay showed that the novel CqFG-derived oligosaccharides with dp ≥ 8 displayed strong APTT- and TT-prolonging activities, whose anticoagulant characteristics differ from various FG-oligosaccharides previously reported. OF2 and OF3 showed potent intrinsic FXase inhibitory activity; the unusual side chains in the oligosaccharides may affect the activity, and their inhibitory activity increased with the increase in *M*_w_. OF2 and OF3 showed negligible bleeding risk, with less bleeding than HP, and LMWHs, indicating that they may be promising, safer anticoagulants. Pure oligosaccharide fragments contained in OF1–OF3 may be charge-isolated by strong anion exchange chromatography further to confirm the connection pattern of the unusual β-d-GlcNAc_6S_ residue and to elucidate the structure–activity relationship of CqFG and its derivatives in the future.

## Figures and Tables

**Table 1:** ^1^H/^13^C NMR chemical shift assignments of OF1.

Residues	H/C	Chemical Shifts (δ, ppm) ^a^								
		1	2	3	4	5	6	7	8	Connection Patterns
F_a1_	H	5.65	*4.51*	4.15	*4.88*	4.96	1.38			
α-l-Fuc_2S4S_	C	100.06	*78.61*	70.07	*84.41*	69.50	19.52			F_a1_1-U3
F_b_	H	5.60	*4.58*	*4.96*	*4.70*	4.89	1.37			
α-l-Fuc_2S3S4S_	C	98.21	*78.61*	*82.69*	*84.29*	70.19	19.52			F_b_1-dU_3_3
F_a2_	H	5.56	*4.50*	4.14	*4.74*	4.72	1.36			
α-l-Fuc_2S4S_	C	100.06	*78.61*	70.07	*84.41*	69.29	19.52			F_a2_1-rU3
F_c_	H	5.54	*4.49*	**3.92**	*4.31*	4.50	1.32			
α-l-Fuc_2S4S_	C	100.06	*78.48*	**76.07**	*73.29*	70.74	19.52			F_c_1-dU_1_3
F_a3_	H	5.50	*4.43*	4.11	*4.70*	4.59	1.32			
α-l-Fuc_2S4S_	C	100.06	*78.48*	70.07	*84.41*	70.36	19.52			F_a3_1-dU_1/2_3
F_d_	H	5.45	*4.40*	4.09	*4.18*	4.23	1.25			
α-l-Fuc_2S_	C	100.06	*78.48*	70.07	73.64	70.74	18.55			F_d_1-dU_1_3
F_a4_	H	5.38	*4.47*	4.08	*4.69*	4.83	1.32			
α-l-Fuc_2S4S_	C	102.29	*78.73*	70.07	*84.16*	70.36	19.52			F_a4_1-dU_1/2_3
F_e_	H	5.24	*3.92*	*4.56*	4.17	4.38	1.30			
α-l-Fuc_3S_	C	101.92	*69.56*	*81.51*	73.64	70.74	19.39			F_e_1-rU3/dU_2/3_3
F_f_	H	5.20	3.80	4.01	*4.61*	4.43	1.36			
α-l-Fuc_4S_	C	102.29	71.79	71.04	*84.16*	70.49	19.52			F_f_1-dU_1_3
G	H	5.10	3.93	*4.54*	*4.36*	4.14	3.80/3.98			
α-d-Gal_3S4S_	C	105.07	69.81	*75.98*	*80.96*	70.83	63.84			G1-F_c_3
dU_1_	H	4.95	3.89	**4.34/4.42**	5.78					
α-l-Δ^4,5^GlcA	C	106.12	73.64	**79.66**	110.33	149.76	172.11			dU_1_1-A_2_3
dU_2_	H	4.94	4.01	**4.20/4.33/4.42**	6.06					
α-l-Δ^4,5^GlcA	C	104.40	71.87	**80.88**	108.56	149.76	172.11			dU_2_1-A_1_3
dU_3_	H	4.93	3.91	**4.48**	5.77					
α-l-Δ^4,5^GlcA	C	106.50	73.64	**79.66**	110.33	149.76	172.11			dU_3_1-A_2_3
A_1_	H	4.79	4.10	**4.27**	*4.85*	4.01	*4.17/4.31*		2.06	
β-d-GalNAc_4S6S_	C	104.52	54.41	**79.41**	*79.62*	75.75	*70.78*	177.98	25.96	A_1_1-U4
U		4.62	3.61	**3.75**	**4.07**	3.77				
β-d-GlcA		99.52	77.98	**80.71**	**82.01**	75.64	179.21			
A_2_	H	4.59	4.09	**4.17**	*4.98*	4.09	*4.17/4.31*		2.06	
β-d-GalNAc_4S6S_	C	103.22	54.83	**80.84**	*79.66*	75.62	*70.78*	*177.98*	25.96	A_2_1-rU4
rU	H	3.78/3.84	3.91	**3.95**	**4.08**	4.78				
β-d-GlcA-ol	C	65.68	72.68	**79.11**	**81.72**	75.96	177.21			

^a^ Values in boldface and italic font indicate glycosylated positions and sulfated positions, respectively.

**Table 2:** Anticoagulant activity of OF1–OF3, and their effects on coagulation factors (*n* = 2).

Sample	APTT ^a^ (μg/mL)	TT ^a^ (μg/mL)	PT ^a^ (μg/mL)	Anti-FXase ^b^ (ng/mL)
HP	0.52 ± 0.18	0.22 ± 0.02	5.11 ± 0.10	18.58 ± 1.44
LMWH	3.28 ± 0.12	1.09 ± 0.10	>128	78.76 ± 10.70
CqFG	2.24 ± 0.07	1.81 ± 0.04	40.73 ± 0.83	45.95 ± 7.02
dCqFG	3.55 ± 0.08	49.90 ± 1.11	>128	51.22 ± 5.68
OF1	34.95 ± 2.22	>128	>128	>2000
OF2	4.88 ± 0.29	100.76 ± 0.89	>128	274.61 ± 25.30
OF3	3.02 ± 0.61	45.02 ± 4.50	>128	136.43 ± 18.65

^a^ The concentration required to double the APTT, TT, or PT of human plasma; ^b^ IC_50_ value, the concentration of each agent required to inhibit 50% of protease activity.

## Data Availability

The original data presented in the study are included in the article/Supplementary Material; further inquiries can be directed to the corresponding author.

## Supplementary Materials

The following supporting information can be downloaded at https://www.mdpi.com/article/10.3390/md23020064/s1. Figure S1: ^1^H NMR spectrum of CqFG; Figure S2: ^1^H NMR spectrum of high-molecular-weight polysaccharide component isolated from dCqFG; Figure S3: Negative ESI-MS spectrum of PMP-labeled acidolysis-resistant disaccharide from CqFG (peak marked x in Figure 1E); Figure S4: ^1^H (A), ^13^C (B), and DEPT-135 (C) NMR spectra of OF1; Figure S5: ^1^H-^1^H TOCSY spectrum of OF1; Figure S6: ^1^H-^13^C HSQC-TOCSY spectrum of OF1; Figure S7: ^1^H (A), ^13^C (B), and DEPT-135 (C) NMR spectra of OF2; Figure S8: ^1^H-^1^H COSY of OF2; Figure S9: ^1^H-^1^H TOCSY spectrum of OF2; Figure S10: ^1^H-^13^C HSQC spectrum of OF2; Figure S11: ^1^H (A), ^13^C (B), and DEPT-135 (C) NMR spectra of OF3; Figure S12: ^1^H-^13^C HMBC spectrum of OF3; Figure S13: Effects of CqFG, dCqFG, and OF1–OF3 on APTT (A), TT (B), and PT (C) of human standard plasma; Figure S14: Effects of CqFG, dCqFG, and OF1–OF3 on intrinsic FXase; Table S1: ^1^H/^13^C NMR chemical shift assignments of OF2 (δ, ppm); Table S2: ^1^H and ^13^C chemical shift assignments of OF3 (δ, ppm).

## References

[1] Xu Y., Masuko S., Takieddin M., Xu H., Liu R., Jing J., Mousa S.A., Linhardt R.J., Liu J. (2011). Chemoenzymatic Synthesis of Homogeneous Ultralow Molecular Weight Heparins. Science.

[2] Xu Y., Chandarajoti K., Zhang X., Pagadala V., Dou W., Hoppensteadt D.M., Sparkenbaugh E.M., Cooley B., Daily S., Key N.S. (2017). Synthetic Oligosaccharides Can Replace Animal-Sourced Low–Molecular Weight Heparins. Sci. Transl. Med..

[3] Caputo H.E., Straub J.E., Grinstaff M.W. (2019). Design, Synthesis, and Biomedical Applications of Synthetic Sulphated Polysaccharides. Chem. Soc. Rev..

[4] Tang H., Huang J., Yuan Q., Lv K., Ma H., Li T., Liu Y., Mi S., Zhao L. (2023). A Regular Chlorella Mannogalactan and Its Sulfated Derivative as a Promising Anticoagulant: Structural Characterization and Anticoagulant Activity. Carbohydr. Polym..

[5] Lin L., Zhao L., Gao N., Yin R., Li S., Sun H., Zhou L., Zhao G., Purcell S.W., Zhao J. (2020). From Multi-Target Anticoagulants to DOACs, and Intrinsic Coagulation Factor Inhibitors. Blood Rev..

[6] Li H., Yuan Q., Lv K., Ma H., Gao C., Liu Y., Zhang S., Zhao L. (2021). Low-Molecular-Weight Fucosylated Glycosaminoglycan and Its Oligosaccharides from Sea Cucumber as Novel Anticoagulants: A Review. Carbohydr. Polym..

[7] Pomin V.H. (2014). Holothurian Fucosylated Chondroitin Sulfate. Mar. Drugs.

[8] Zhao L., Lai S., Huang R., Wu M., Gao N., Xu L., Qin H., Peng W., Zhao J. (2013). Structure and Anticoagulant Activity of Fucosylated Glycosaminoglycan Degraded by Deaminative Cleavage. Carbohydr. Polym..

[9] Zhao L., Wu M., Xiao C., Yang L., Zhou L., Gao N., Li Z., Chen J., Chen J., Liu J. (2015). Discovery of an Intrinsic Tenase Complex Inhibitor: Pure Nonasaccharide from Fucosylated Glycosaminoglycan. Proc. Natl. Acad. Sci. USA.

[10] Yin R., Zhou L., Gao N., Li Z., Zhao L., Shang F., Wu M., Zhao J. (2018). Oligosaccharides from Depolymerized Fucosylated Glycosaminoglycan: Structures and Minimum Size for Intrinsic Factor Xase Complex Inhibition. J. Biol. Chem..

[11] Pan Y., Sun H., Gu X., Li S., Yang S., Zhang L., Mao H., Wang P., Yang S., Yin R. (2025). Oligosaccharide-Assisted Resolution of Holothurian Fucosylated Chondroitin Sulfate for Fine Structure and P-Selectin Inhibition. Carbohydr. Polym..

[12] Yin R., Zhou L., Gao N., Lin L., Sun H., Chen D., Cai Y., Zuo Z., Hu K., Huang S. (2021). Unveiling the Disaccharide-Branched Glycosaminoglycan and Anticoagulant Potential of Its Derivatives. Biomacromolecules.

[13] Li S., Zhong W., Pan Y., Lin L., Cai Y., Mao H., Zhang T., Li S., Chen R., Zhou L. (2021). Structural Characterization and Anticoagulant Analysis of the Novel Branched Fucosylated Glycosaminoglycan from Sea Cucumber Holothuria Nobilis. Carbohydr. Polym..

[14] Mao H., Cai Y., Li S., Sun H., Lin L., Pan Y., Yang W., He Z., Chen R., Zhou L. (2020). A New Fucosylated Glycosaminoglycan Containing Disaccharide Branches from Acaudina Molpadioides: Unusual Structure and Anti-Intrinsic Tenase Activity. Carbohydr. Polym..

[15] Yuan Q., Li H., Wang Q., Sun S., Fang Z., Tang H., Shi X., Wen J., Huang L., Bai M. (2022). Deaminative-Cleaved, S. Monotuberculatus Fucosylated Glycosaminoglycan: Structural Elucidation and Anticoagulant Activity. Carbohydr. Polym..

[16] Lan D., Zhang J., Shang X., Yu L., Xu C., Wang P., Cui L., Cheng N., Sun H., Ran J. (2023). Branch Distribution Pattern and Anticoagulant Activity of a Fucosylated Chondroitin Sulfate from Phyllophorella Kohkutiensis. Carbohydr. Polym..

[17] Yin R., Pan Y., Cai Y., Yang F., Gao N., Ruzemaimaiti D., Zhao J. (2022). Re-Understanding of Structure and Anticoagulation: Fucosylated Chondroitin Sulfate from Sea Cucumber Ludwigothurea Grisea. Carbohydr. Polym..

[18] Zeng L., Wen J., Lin H., Fan S., Sun Y., Yang C., Zhao J., Li X. (2020). The Complete Mitochondrial Genome of Colochirus Quadrangularis (Dendrochirotida, Cucumariidae). Mitochondrial DNA Part. B.

[19] Gao N., Chen R., Mou R., Xiang J., Zhou K., Li Z., Zhao J. (2020). Purification, Structural Characterization and Anticoagulant Activities of Four Sulfated Polysaccharides from Sea Cucumber Holothuria Fuscopunctata. Int. J. Biol. Macromol..

[20] Qiu P., Wu F., Yi L., Chen L., Jin Y., Ding X., Ouyang Y., Yao Y., Jiang Y., Zhang Z. (2020). Structure Characterization of a Heavily Fucosylated Chondroitin Sulfate from Sea Cucumber (H. Leucospilota) with Bottom-up Strategies. Carbohydr. Polym..

[21] Santos G.R.C., Porto A.C.O., Soares P.A.G., Vilanova E., Mourão P.A.S. (2017). Exploring the Structure of Fucosylated Chondroitin Sulfate through Bottom-up Nuclear Magnetic Resonance and Electrospray Ionization-High-Resolution Mass Spectrometry Approaches. Glycobiology.

[22] Kariya Y., Watabe S., Hashimoto K., Yoshida K. (1990). Occurrence of Chondroitin Sulfate E in Glycosaminoglycan Isolated from the Body Wall of Sea Cucumber Stichopus Japonicus. J. Biol. Chem..

[23] Guan R., Peng Y., Zhou L., Zheng W., Liu X., Wang P., Yuan Q., Gao N., Zhao L., Zhao J. (2019). Precise Structure and Anticoagulant Activity of Fucosylated Glycosaminoglycan from Apostichopus Japonicus: Analysis of Its Depolymerized Fragments. Mar. Drugs.

[24] Sun H., Gao N., Ren L., Liu S., Lin L., Zheng W., Zhou L., Yin R., Zhao J. (2020). The Components and Activities Analysis of a Novel Anticoagulant Candidate DHG-5. Eur. J. Med. Chem..

[25] Ustyuzhanina N.E., Bilan M.I., Anisimova N.Y., Dmitrenok A.S., Tsvetkova E.A., Kiselevskiy M.V., Nifantiev N.E., Usov A.I. (2022). Depolymerization of a Fucosylated Chondroitin Sulfate from Cucumaria Japonica: Structure and Activity of the Product. Carbohydr. Polym..

[26] Ustyuzhanina N.E., Bilan M.I., Dmitrenok A.S., Shashkov A.S., Ponce N.M.A., Stortz C.A., Nifantiev N.E., Usov A.I. (2020). Fucosylated Chondroitin Sulfate from the Sea Cucumber Hemioedema Spectabilis: Structure and Influence on Cell Adhesion and Tubulogenesis. Carbohydr. Polym..

[27] Li Q., Cai C., Chang Y., Zhang F., Linhardt R.J., Xue C., Li G., Yu G. (2018). A Novel Structural Fucosylated Chondroitin Sulfate from Holothuria Mexicana and Its Effects on Growth Factors Binding and Anticoagulation. Carbohydr. Polym..

[28] Wu M., Wen D., Gao N., Xiao C., Yang L., Xu L., Lian W., Peng W., Jiang J., Zhao J. (2015). Anticoagulant and Antithrombotic Evaluation of Native Fucosylated Chondroitin Sulfates and Their Derivatives as Selective Inhibitors of Intrinsic Factor Xase. Eur. J. Med. Chem..

[29] Wu M., Huang R., Wen D., Gao N., He J., Li Z., Zhao J. (2012). Structure and Effect of Sulfated Fucose Branches on Anticoagulant Activity of the Fucosylated Chondroitin Sulfate from Sea Cucumber Thelenata Ananas. Carbohydr. Polym..

[30] Zhou L., Gao N., Sun H., Xiao C., Yang L., Lin L., Yin R., Li Z., Zhang H., Ji X. (2020). Effects of Native Fucosylated Glycosaminoglycan, Its Depolymerized Derivatives on Intrinsic Factor Xase, Coagulation, Thrombosis, and Hemorrhagic Risk. Thromb. Haemost..

[31] Cai Y., Yang W., Li X., Zhou L., Wang Z., Lin L., Chen D., Zhao L., Li Z., Liu S. (2019). Precise Structures and Anti-Intrinsic Tenase Complex Activity of Three Fucosylated Glycosaminoglycans and Their Fragments. Carbohydr. Polym..

[32] Gao N., Lu F., Xiao C., Yang L., Chen J., Zhou K., Wen D., Li Z., Wu M., Jiang J. (2015). β-Eliminative Depolymerization of the Fucosylated Chondroitin Sulfate and Anticoagulant Activities of Resulting Fragments. Carbohydr. Polym..

[33] Yuan Q., Liang R., Lv K., Shi X., Leng J., Liu Y., Xiao J., Zhang L., Zhao L. (2024). Structural Characterization of a Chlorella Heteropolysaccharide by Analyzing Its Depolymerized Product and Finding an Inducer of Human Dendritic Cell Maturation. Carbohydr. Polym..

[34] Xiao C., Zhao L., Gao N., Wu M., Zhao J. (2019). Nonasaccharide Inhibits Intrinsic Factor Xase Complex by Binding to Factor IXa and Disrupting Factor IXa-Factor VIIIa Interactions. Thromb. Haemost..

