# Supplementary material for: Structural Characterization and Anticoagulant Potential of Colochirus quadrangularis Fucosylated Glycosaminoglycan 5−12 Oligomers with Unusual Branches
Source: Mar Drugs. 2025 Feb 1;23(2):64. doi: 10.3390/md23020064 (PMC11857587; doi:10.3390/md23020064)
Supplement: Supplementary file 1 [file marinedrugs-23-00064-s001.zip › marinedrugs-3439599-supplementary.pdf]

Supplementary Materials

Structural characterization and anticoagulant potential of Colochirus quadrangularis fucosylated glycosaminoglycan 5–12 oligomers with unusual branches

Xuedong Zhang [1,†], Guangwei Yan [2,†], Xinming Liu [1,†], Jiewen Fu [1], Xiang Shi [1,2], Pei Cao [1], Yuqian Sun [3], Shengping Zhong [1], Jiale Nong [1], Peiqi Jiang [1], Yonghong Liu [1], Baoshun Zhang [2,*], Qingxia Yuan [1,*], Longyan Zhao [1,*]

[1] Guangxi Key Laboratory of Marine Drugs, Institute of Marine Drugs, Guangxi University of Chinese Medicine, Nanning 530200, China

[2] College of Pharmaceutical Sciences, Southwest University, Chongqing 400716, PR China.

[3] Instrumentation and Service Center for Molecular Sciences, Westlake University, Hangzhou 310024, China

[*]Correspondence: zbs360@swu.edu.cn (B. Zhang); qingxiayuan@163.com (Q. Yuan); zhaolongyan-dra@163.com (L. Zhao).

[†] These authors contributed equally to this work.

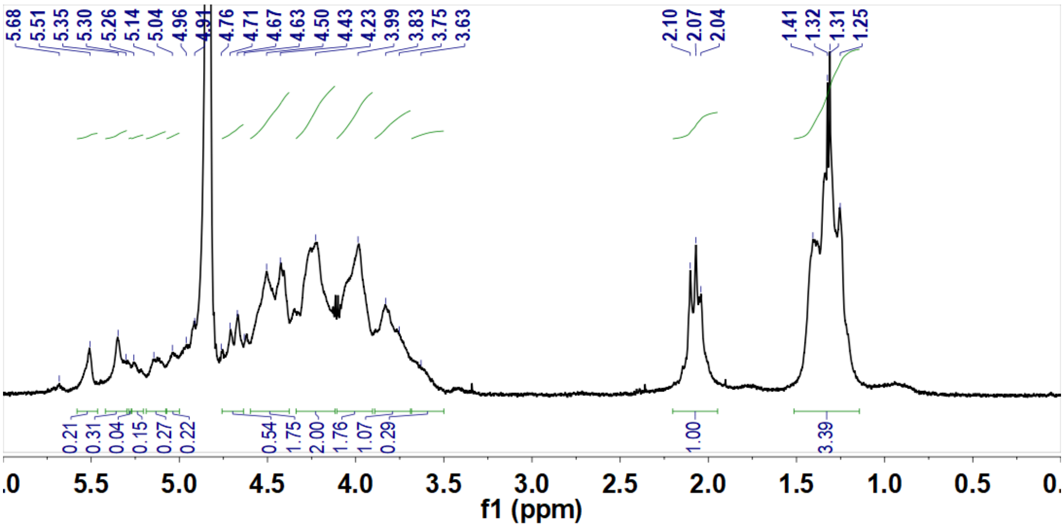

Figure S1. ^{1}H NMR spectrum of CqFG

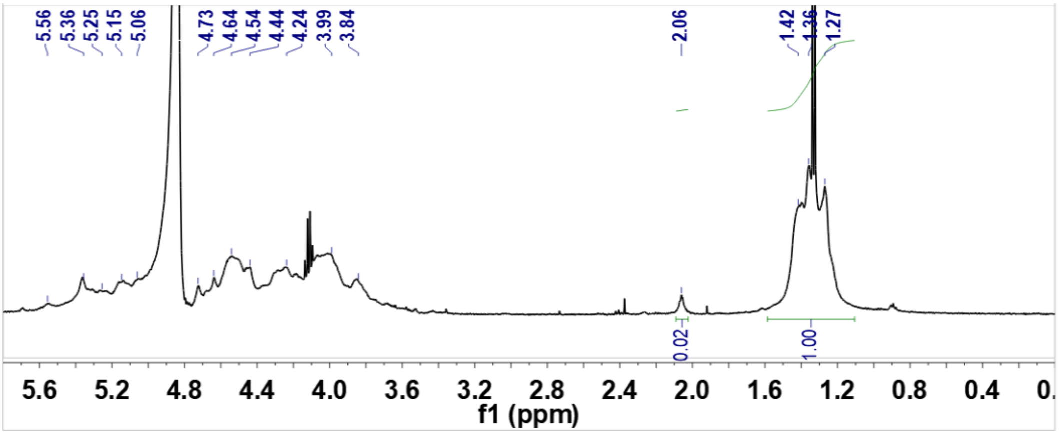

Figure S2. ^{1}H NMR spectrum of high-molecular-weight polysaccharide component isolated from dCqFG

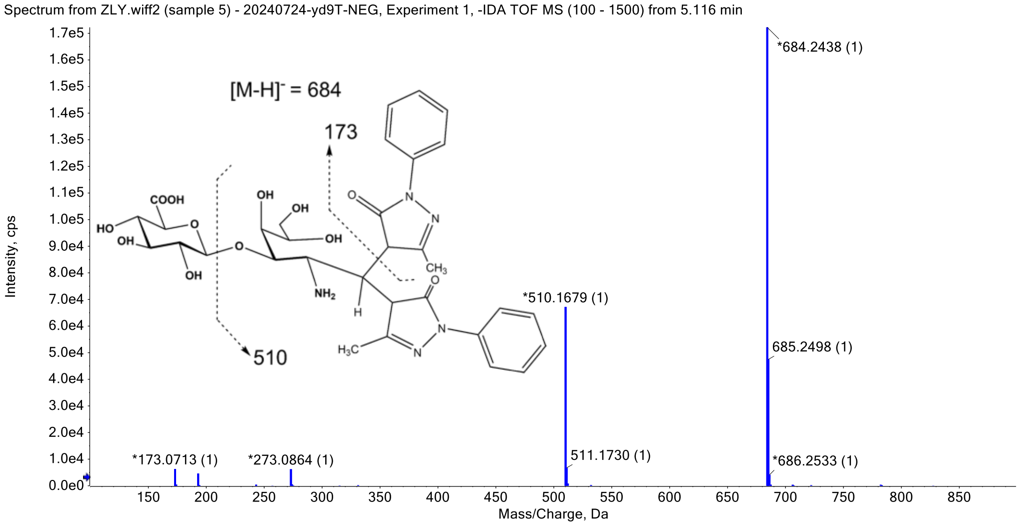

Figure S3. Negative ESI-MS spectrum of PMP-labeled acidolysis-resistant disaccharide from CqFG (peak marked x in Fig. 1E).

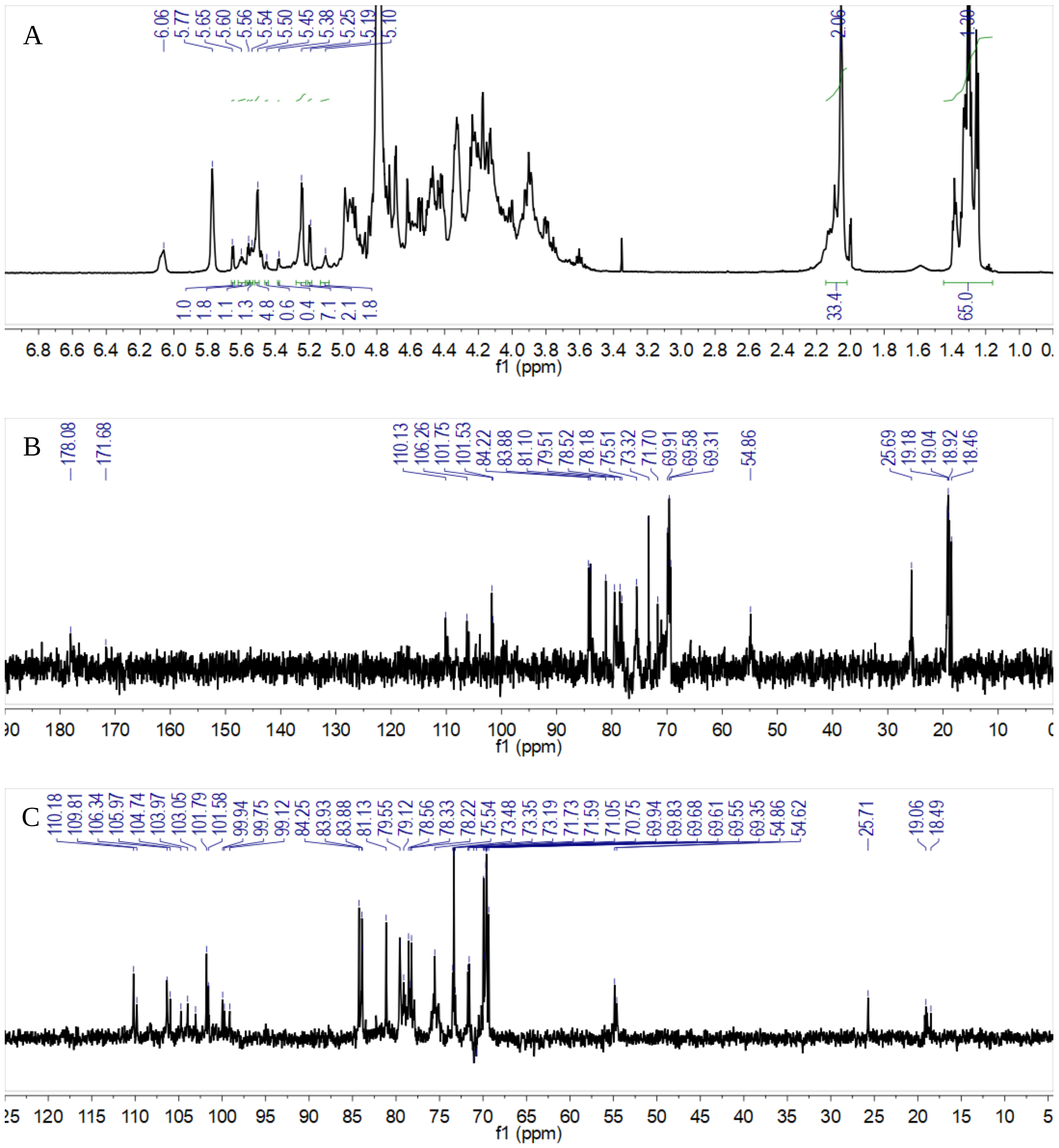

Figure S4. ^{1}H (A), ^{13}C (B), and DEPT-135 (C) NMR spectra of OF1.

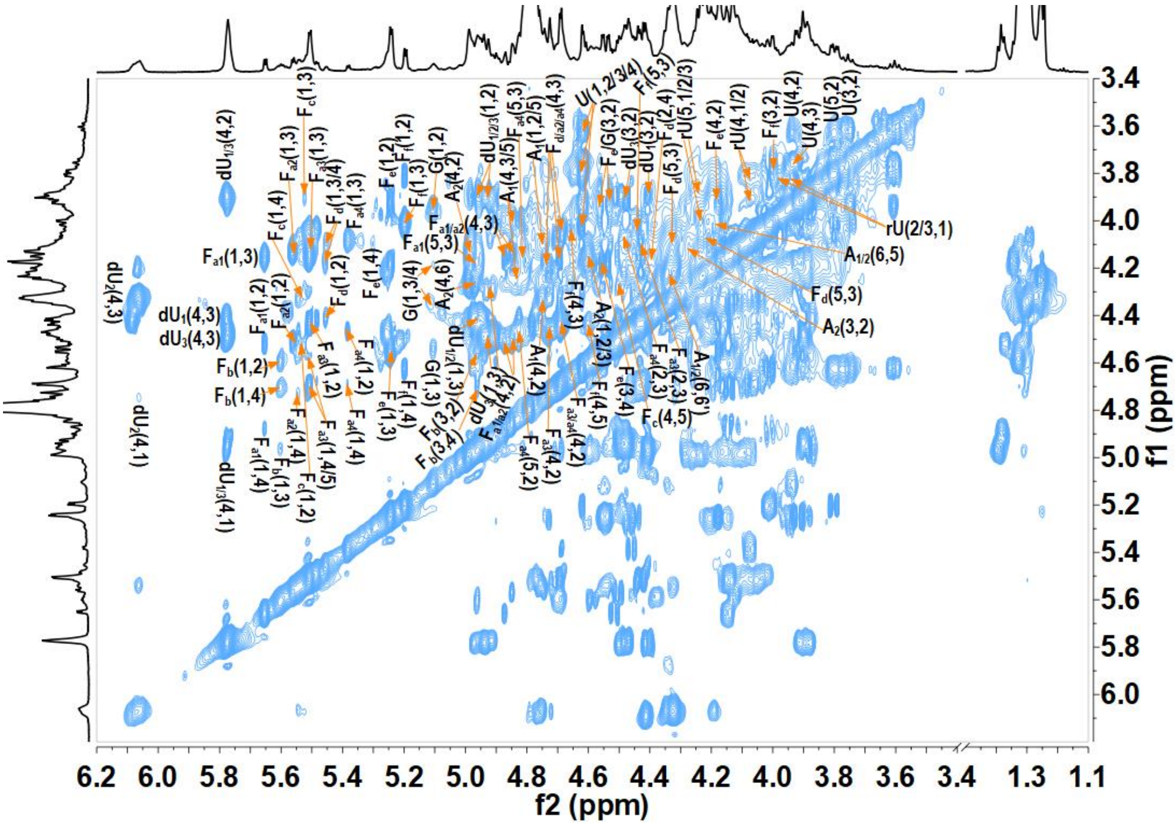

Figure S5. ${}^{1}H$-${}^{1}H$ TOCSY spectrum of OF1.

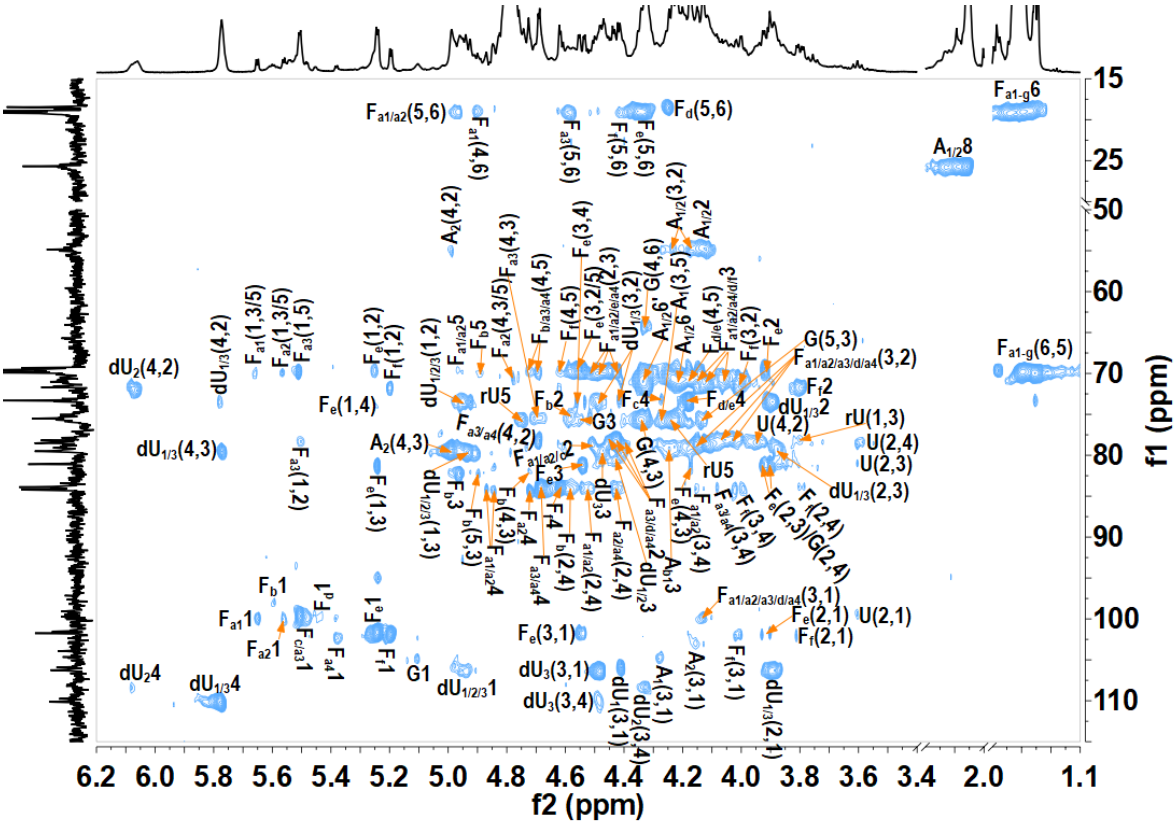

Figure S6. ${}^{1}H$-${}^{13}C$ HSQC-TOCSY spectrum of OF1.

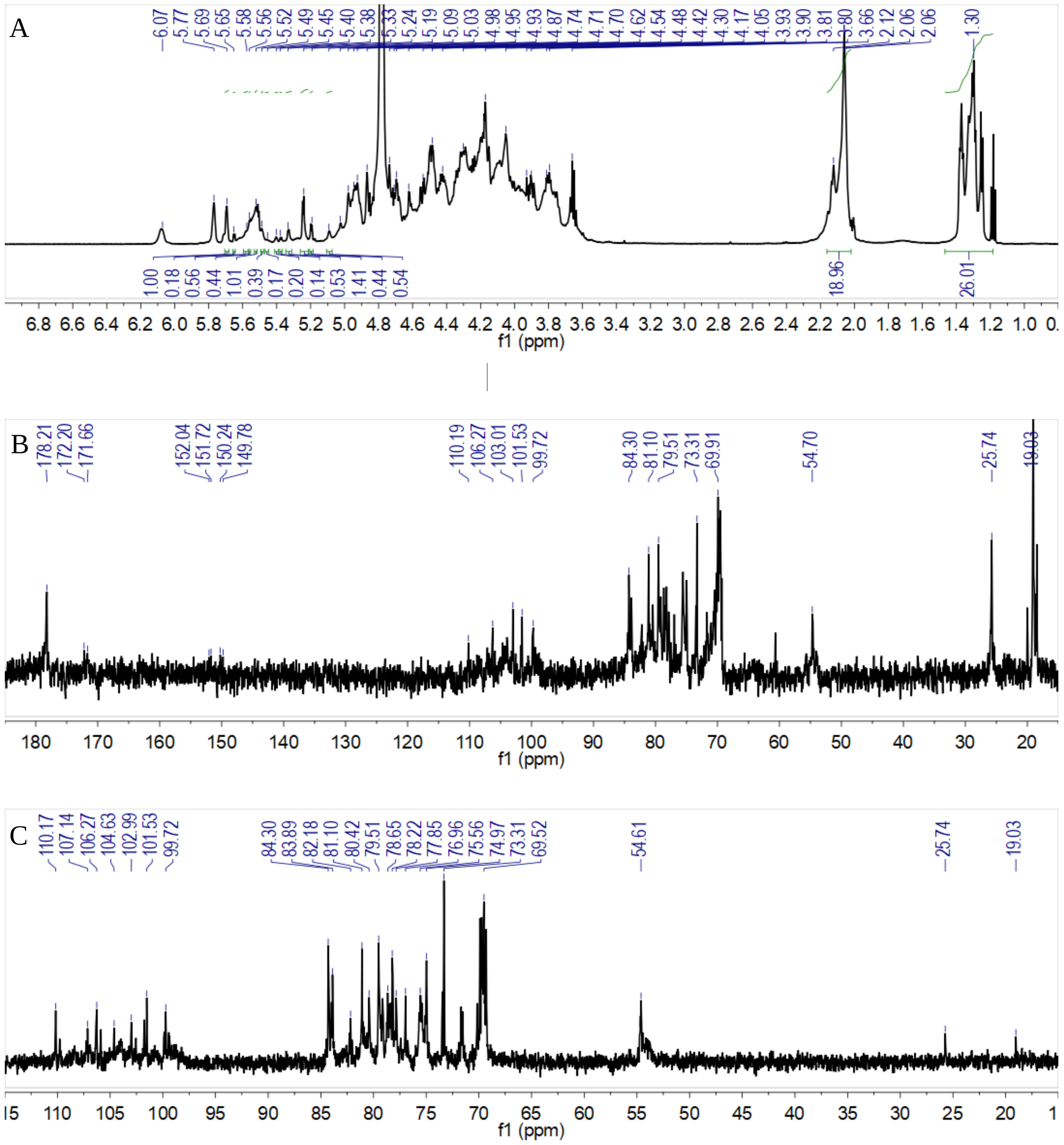

Figure S7. ^{1}H (A), ^{13}C (B), and DEPT-135 (C) NMR spectra of OF2.

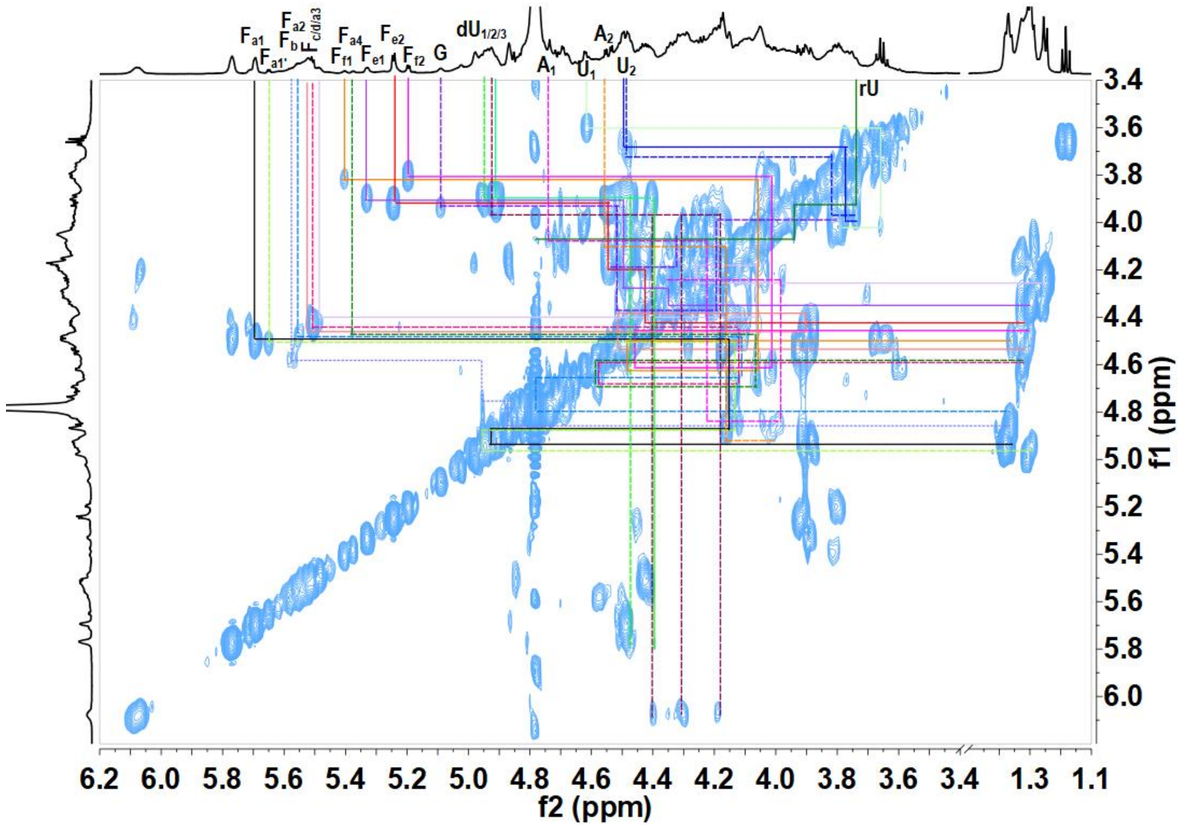

Figure S8. ^{1}H-^{1}H COSY of OF2.

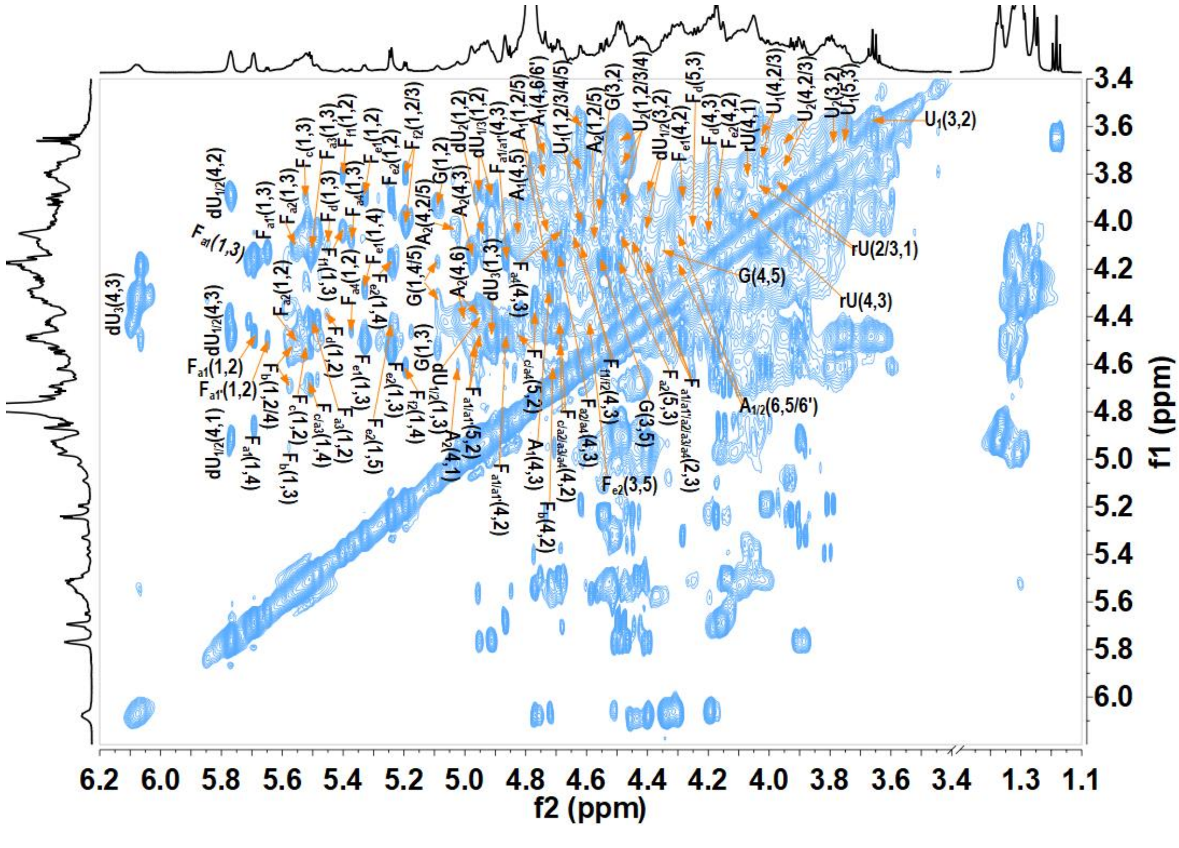

Figuure S9. ^{1}H-^{1}H TOCSY spectrum of OF2.

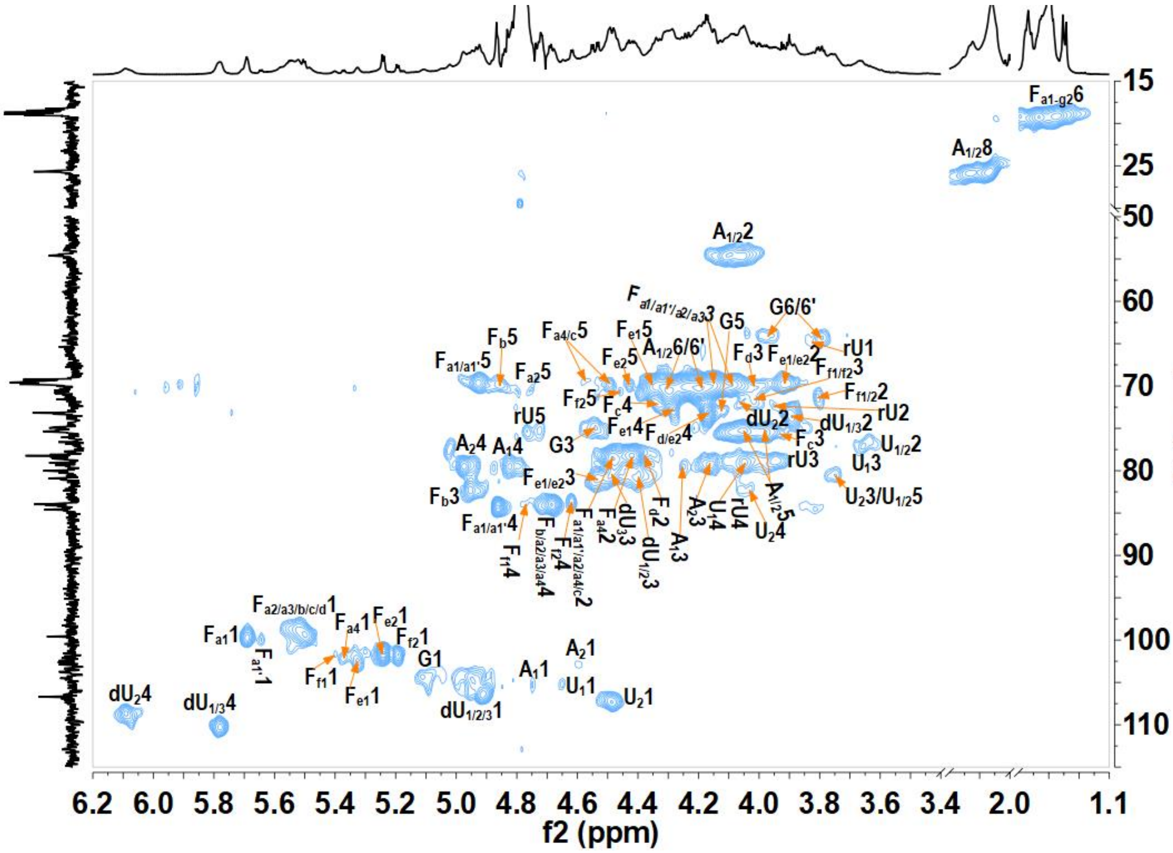

Figure S10. ^1H-^{13}C HSQC spectrum of OF2.

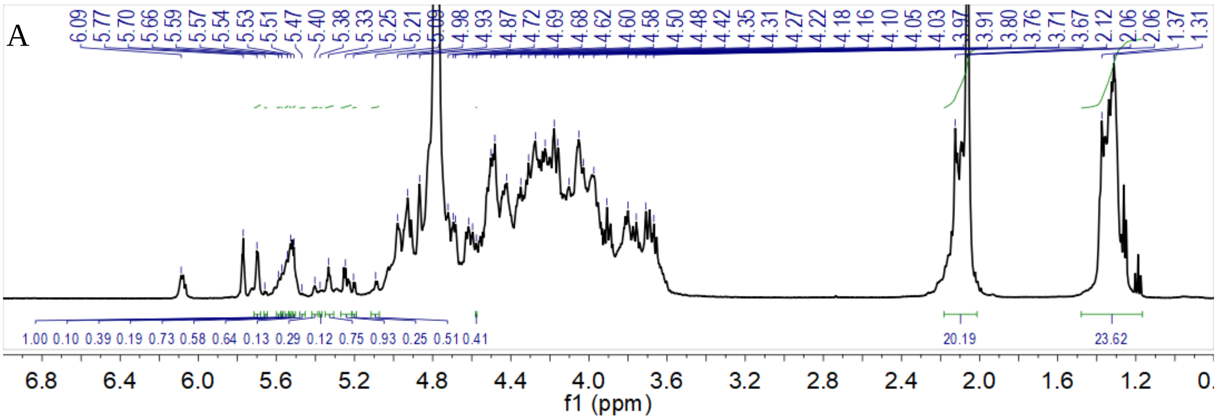

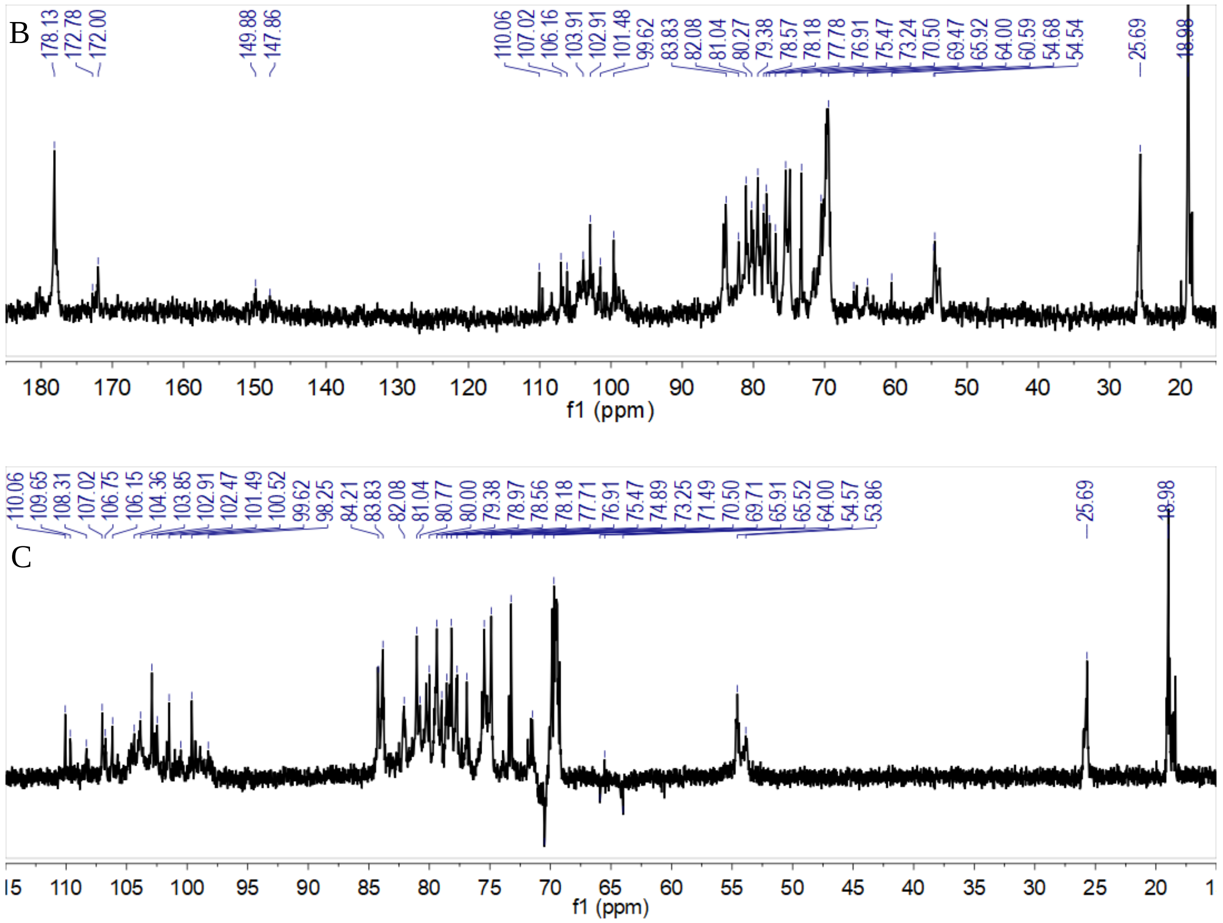

Figure S11. ^{1}H (A), ^{13}C (B), and DEPT-135 (C) NMR spectra of OF3.

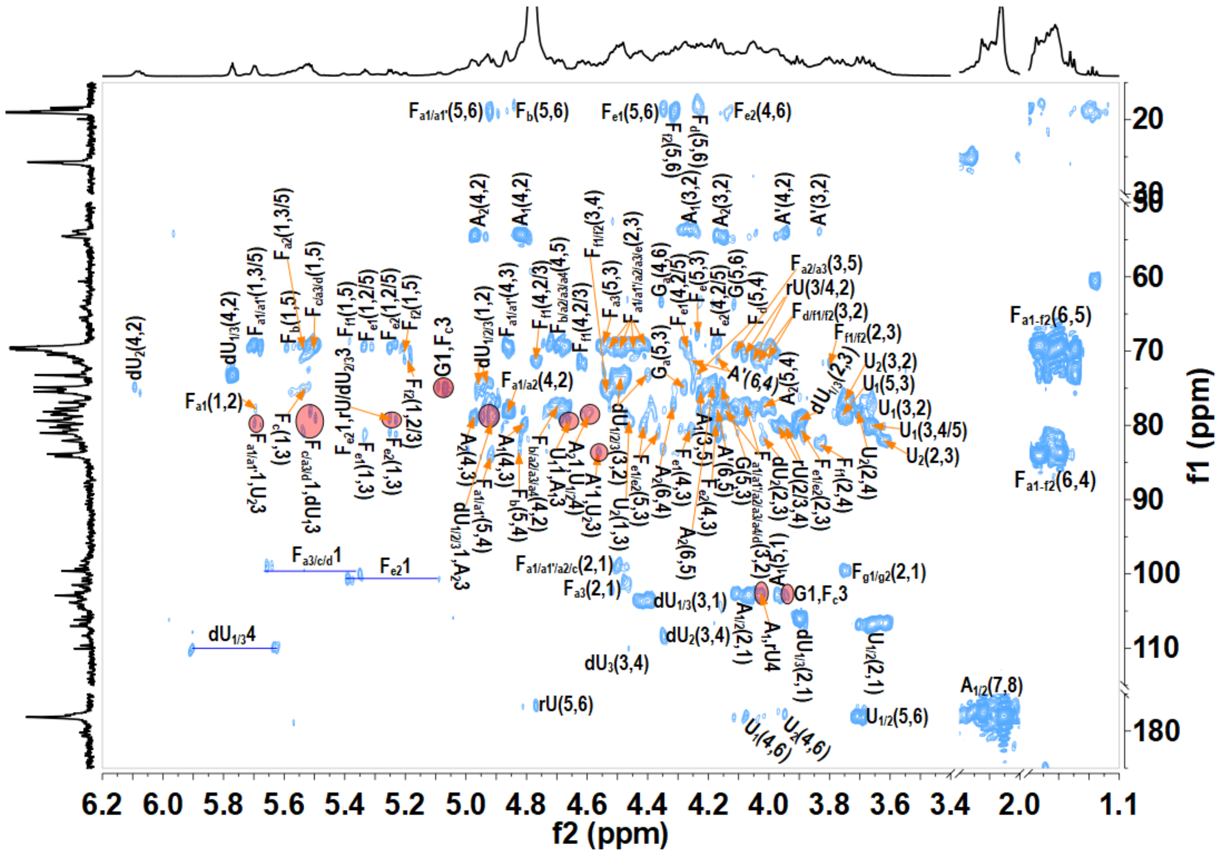

Figure S12. ^{1}H-^{13}C HMBC spectrum of OF3.

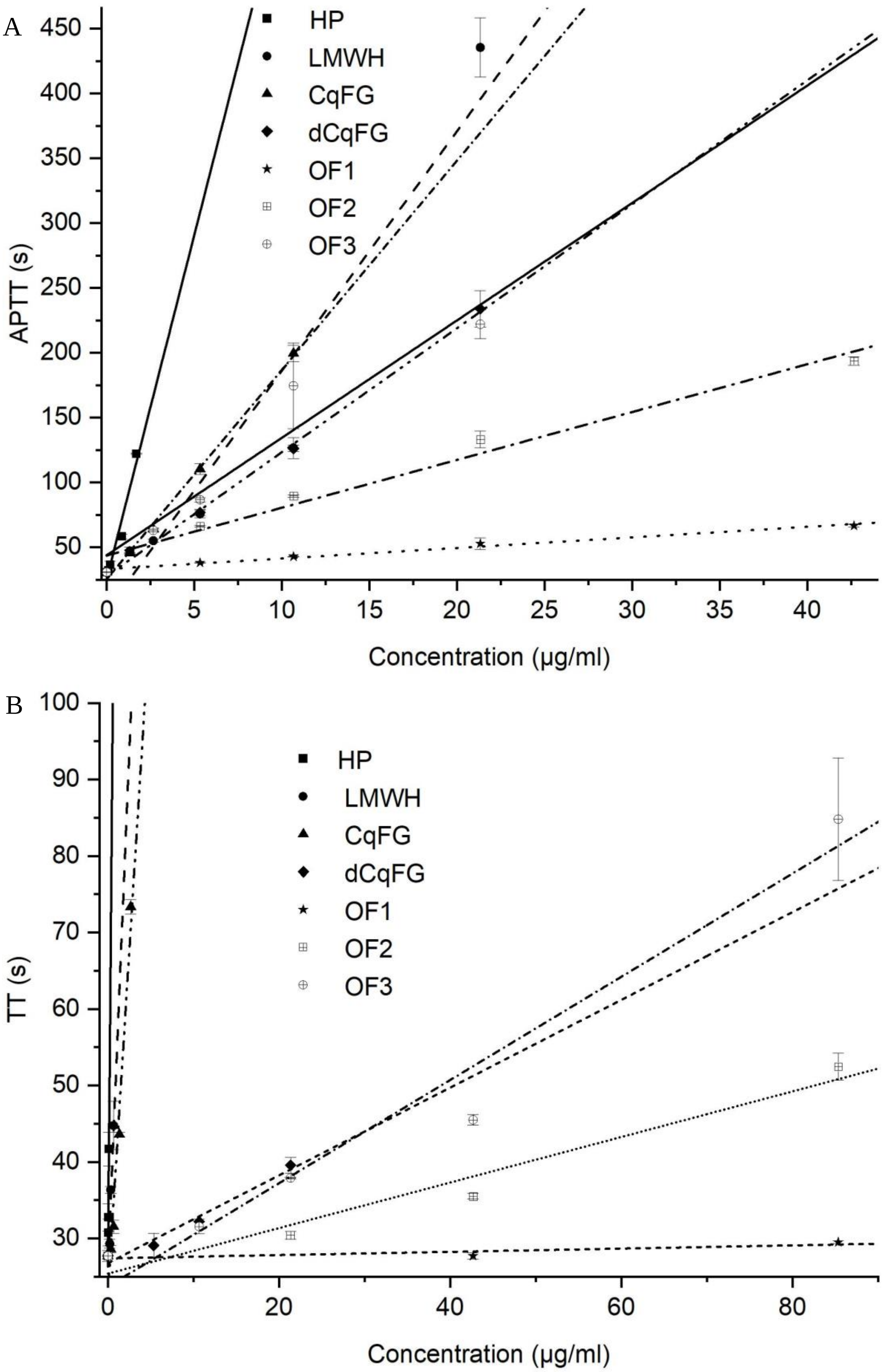
A
APTT (s)
Concentration (μg/ml)
HP
LMWH
CqFG
dCqFG
OF1
OF2
OF3
B
TT (s)
Concentration (μg/ml)
HP
LMWH
CqFG
dCqFG
OF1
OF2
OF3

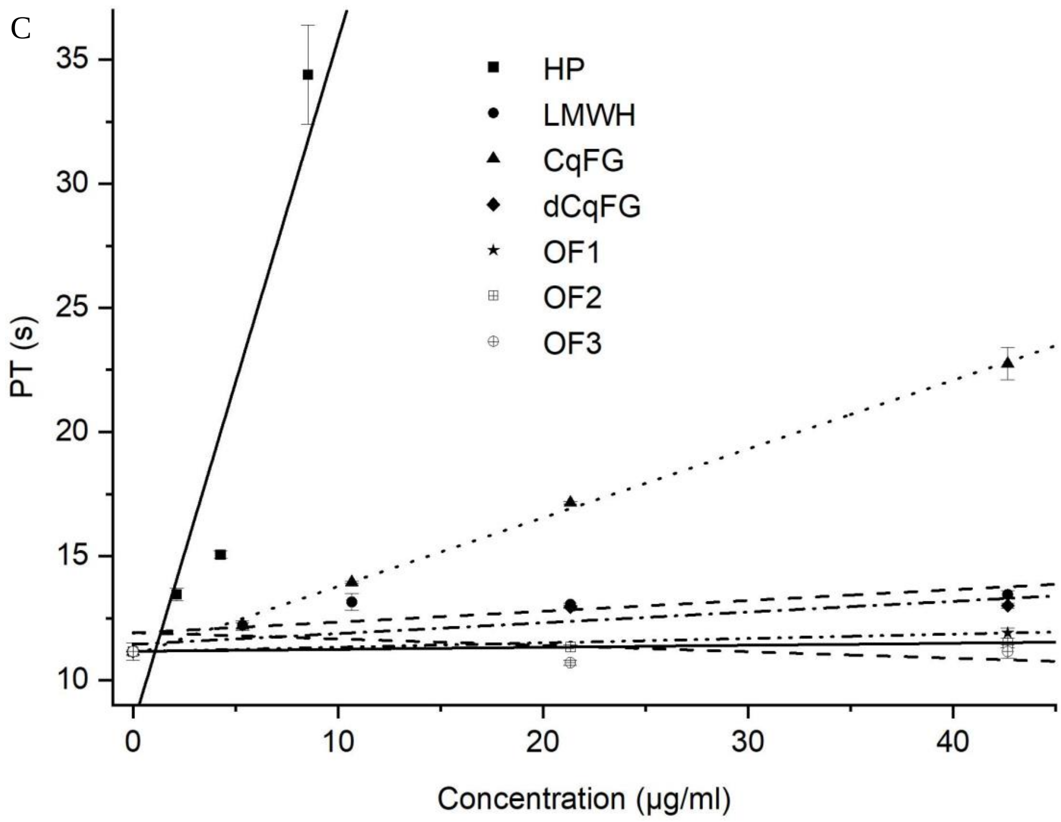

Figure S13. Effects of CqFG, dCqFG, and **OF1**–**OF3** on APTT (A), TT (B) and PT (C) of human standard plasma.

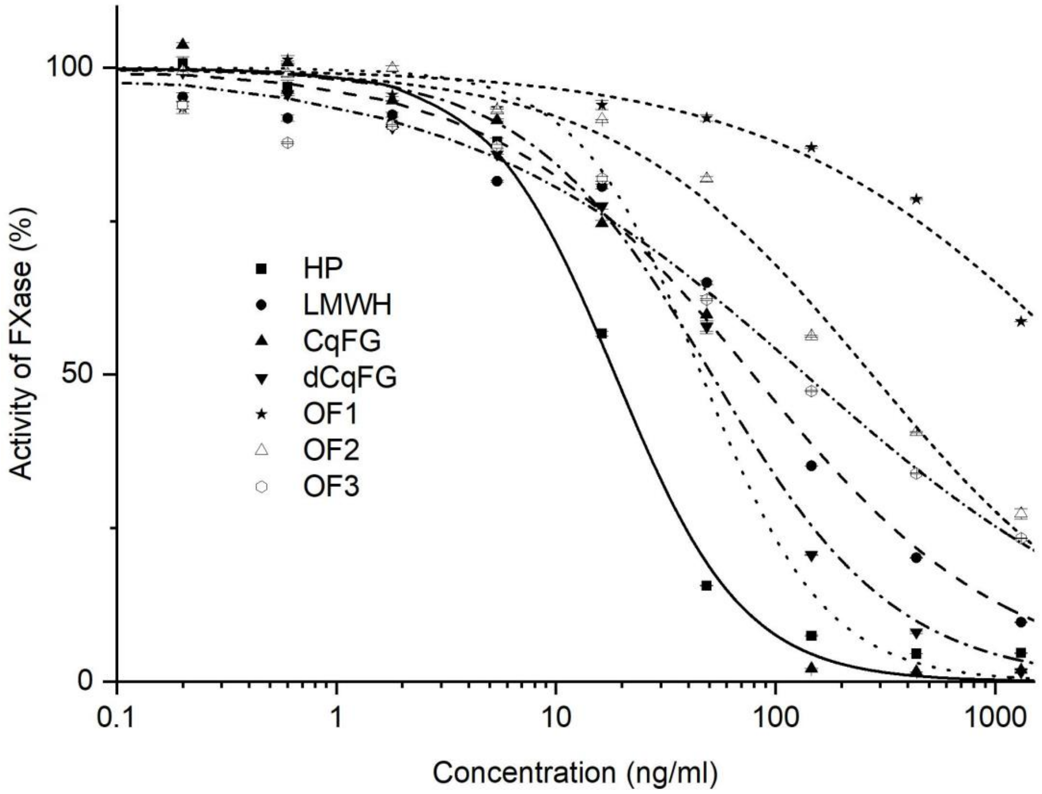

Figure S14. Effects of CqFG, dCqFG, and **OF1**–**OF3** on intrinsic FXase

Table S1. ^{1}H/^{13}C NMR chemical shift assignments of **OF2** (δ, ppm).

Residues	H/C	Chemical shifts (δ, ppm) [a]								
		1	2	3	4	5	6	7	8	Connection patterns
F_{a1}	H	5.69	*4.49*	4.17	*4.86*	4.92	1.36			
α-L-Fuc$_{2S4S}$	C	99.77	*78.48*	70.15	*84.45*	69.73	19.44			F_{a1}1-$U_2$3
$F_{a1'}$	H	5.65	*4.51*	4.13	*4.87*	4.96	1.33			
α-L-Fuc$_{2S4S}$	C	100.06	*78.40*	70.15	*84.45*	69.56	19.44			$F_{a1'}$1-$U_2$3
F_b	H	5.57	*4.59*	*4.95*	*4.70*	4.86	1.36			
α-L-Fuc$_{2S3S4S}$	C	98.55	*78.57*	*82.31*	*84.12*	69.98	19.10			F_b1-d$U_2$3
F_{a2}	H	5.56	*4.50*	4.12	*4.65*	4.75	1.37			
α-L-Fuc$_{2S4S}$	C	99.01	*78.56*	69.98	*84.75*	69.98	19.10			F_{a2}1-rU3
F_c	H	5.53	*4.49*	**3.94**	*4.31*	4.50	1.31			
α-L-Fuc$_{2S4S}$	C	99.43	*78.23*	**75.77**	*73.29*	70.74	19.10			F_c1-d$U_1$3
F_{a3}	H	5.50	4.43	4.10	*4.68*	4.58	1.32			
α-L-Fuc$_{2S4S}$	C	99.43	*78.23*	70.04	*84.12*	70.36	19.10			F_{a3}1-d$U_{1/2}$3
F_d	H	5.45	4.39	*4.02*	*4.18*	4.25	1.25			
α-L-Fuc$_{2S}$	C	99.43	*78.23*	69.98	*73.64*	70.74	19.10			F_d1-d$U_1$3
F_{f1}	H	5.41	3.80	4.04	*4.77*	4.32	1.35			
α-L-Fuc$_{4S}$	C	101.75	71.41	71.58	*84.05*	69.69	19.52			F_{f1}1-$U_1$3
F_{a4}	H	5.38	4.46	4.07	*4.69*	4.58	1.31			
α-L-Fuc$_{2S4S}$	C	102.04	*78.66*	69.98	*84.12*	70.36	19.52			F_{a4}1-d$U_{1/2}$3
F_{e1}	H	5.33	3.89	*4.50*	4.28	4.37	1.30			
α-L-Fuc$_{3S}$	C	102.55	69.73	*81.38*	73.22	70.15	19.39			F_{e1}1-$U_1$3
F_{e2}	H	5.24	3.91	*4.55*	4.18	4.43	1.31			
α-L-Fuc$_{3S}$	C	101.75	69.73	*81.38*	73.31	69.98	19.39			F_{e2}1-rU/d$U_{2/3}$3
F_{f2}	H	5.20	3.80	4.00	*4.62*	4.46	1.30			
α-L-Fuc$_{4S}$	C	101.92	71.41	71.41	*83.74*	70.23	19.52			F_{f2}1-d$U_1$3
G	H	5.09	3.93	*4.54*	*4.36*	4.18	3.80/3.98			
α-D-Gal$_{3S4S}$	C	104.69	69.81	*75.16*	*80.67*	71.85	64.60			G1-F_c3
dU_1	H	4.96	3.88	**4.35/4.41**	5.78					
α-L-Δ^{4,5}GlcA	C	105.83	73.47	**80.58**	110.29	149.78	171.66			d$U_1$1-$A_2$3
dU_2	H	4.94	4.01	**4.20/4.32/4.42**	6.07/6.09					
α-L-Δ^{4,5}GlcA	C	104.52	72.04	**79.53**	108.56	152.04	172.20			d$U_2$1-$A_2$3
dU_3	H	4.93	3.91	**4.23/4.48**	5.77					
α-L-Δ^{4,5}GlcA	C	106.46	73.47	**80.58**	110.29	149.78	171.66			d$U_3$1-$A_2$3
A_1	H	4.74	4.08	**4.27**	*4.83/4.74*	3.99	*4.17/4.31*		2.06	
β-D-GalNAc$_{4S6S}$	C	105.45	55.39	**79.53**	*79.53*	75.06	*70.78*	177.98	25.83	$A_1$1-rU4
U_1	H	4.65	3.61	**3.66**	**4.03**	3.78				
β-D-GlcA	C	105.20	76.63	**77.43**	**78.94**	80.46	178.21			U_1-$A_2$3
A_2	H	4.59	4.07	**4.17**	*4.98/5.02*	4.06	*4.17/4.35*		2.13	
β-D-GalNAc$_{4S6S}$	C	103.05	54.83	**79.41**	*79.66/77.81*	75.62	*70.78*	177.98	26.00	A_2-$U_{1/2}$4
U_2	H	4.49	3.65	**3.75**	**4.03**	3.75				
β-D-GlcA	C	107.26	76.18	**80.58**	**82.35**	80.46	178.21			U_2-$A_1$3

rU	H	3.73/3.84	3.95	**3.96**	**4.06**	4.77				
β-D-GlcA-ol	C	65.68	72.04	**79.07**	**82.18**	75.07	177.23			

[a] Values in boldface and italic font indicate glycosylated and sulfated positions, respectively.

Table S2. ^{1}H and ^{13}C chemical shift assignments of **OF3** (δ, ppm).

Residues	H/C	Chemical shifts (δ, ppm)								Connection patterns
		1	2	3	4	5	6	7	8	
F_{a1}	H	5.70	*4.49*	4.15	*4.87*	4.92	1.38			
α-L-Fuc_{2S4S}	C	99.47	*78.10*	69.60	*84.08*	69.10	18.68			F_{a1}1-$U_2$3
$F_{a1'}$	H	5.66	*4.51*	4.13	*4.87*	4.93	1.35			
α-L-Fuc_{2S4S}	C	100.06	*78.40*	69.60	*84.08*	69.10	18.68			$F_{a1'}$1-$U_2$3
F_b	H	5.59	*4.58*	*4.93*	*4.76*	4.84	1.37			
α-L-Fuc_{2S3S4S}	C	98.21	*78.57*	*81.85*	*84.03*	69.35	18.68			F_b1-d$U_2$3
F_{a2}	H	5.57	*4.48*	*4.12*	*4.67*	4.79	1.35			
α-L-Fuc_{2S4S}	C	100.06	*78.56*	69.56	*83.61*	69.35	18.68			F_{a2}1-rU3
F_c	H	5.53	4.45	**3.94**	*4.31*	4.50	1.31			
α-L-Fuc_{2S}	C	98.59	*77.76*	**75.77**	*73.29*	70.74	19.10			F_c1-d$U_1$3
F_{a3}	H	5.51	4.43	4.10	*4.68*	4.60	1.35			
α-L-Fuc_{2S4S}	C	99.81	*77.85*	69.56	*83.70*	69.35	18.68			F_{a3}1-d$U_{1/2}$3
F_d	H	5.48	4.40	*4.02*	*4.18*	4.25	1.30			
α-L-Fuc_{2S}	C	99.85	*78.02*	69.43	*73.05*	69.81	19.10			F_d1-d$U_1$3
F_{f1}	H	5.40	3.82	4.05	*4.77*	4.49	1.35			
α-L-Fuc_{4S}	C	101.45	71.20	71.54	*83.61*	69.81	18.68			F_{f1}1-$U_1$3
F_{a4}	H	5.38	4.45	4.07	*4.70*	4.59	1.31			
α-L-Fuc_{2S4S}	C	102.04	*78.66*	69.98	*84.12*	70.36	18.68			F_{a4}1-d$U_{1/2}$3
F_{e1}	H	5.33	3.89	*4.50*	4.28	4.35	1.32			
α-L-Fuc_{3S}	C	102.17	69.14	*81.00*	73.05	69.90	18.68			F_{e1}1-$U_1$3
F_{e2}	H	5.25	3.91	*4.55*	4.17	4.40	1.31			
α-L-Fuc_{3S}	C	101.37	69.14	*81.00*	73.05	69.90	18.68			F_{e2}1-rU/d$U_{2/3}$3
F_{f2}	H	5.21	3.80	4.01	*4.62*	4.32	1.31			
α-L-Fuc_{4S}	C	101.45	71.20	71.54	*83.61*	69.73	18.68			F_{f2}1-d$U_1$3
G	H	5.09	3.93	*4.52*	*4.34*	4.16	3.83/3.96			
α-D-Gal_{3S4S}	C	104.15	69.01	*75.07*	*80.67*	72.17	63.96			G1-F_c3
dU_1	H	4.95	3.90	**4.35/4.41**	5.78					
α-L-$\Delta^{4,5}$GlcA	C	106.04	73.22	**80.67**	109.78	147.86	172.00			d$U_1$1-$A_2$3
dU_2	H	4.94	4.02	**4.26/4.32/4.42**	6.08					
α-L-$\Delta^{4,5}$GlcA	C	104.19	72.65	**81.00**	108.23	149.88	172.78			d$U_2$1-$A_2$3
dU_3	H	4.91	3.91	**4.23/4.49**	5.77					
α-L-$\Delta^{4,5}$GlcA	C	106.04	73.22	**80.67**	109.78	147.86	172.00			d$U_3$1-$A_2$3
A_1	H	4.78	4.04	**4.26**	*4.81*	3.99	*4.17/4.31*		2.06	
β-D-$GalNAc_{4S6S}$	C	103.85	54.04	**79.83**	*79.28*	74.95	*70.78*	177.98	25.83	$A_1$1-rU4
U_1	H	4.62	3.59	**3.68**	**4.03**	3.76				

β-D-GlcA	C	105.20	76.59	**79.87**	**78.77**	79.95	178.21			$U_1$1-$A_2$3
A_2	H	4.60	4.07	**4.18**	*4.98/5.02*	4.02	*4.17/4.35*		2.13	
β-D-GalNAc$_{4S6S}$	C	102.63	54.12	**79.41**	*79.20/77.09*	74.95	*70.78*	177.98	26.00	$A_2$1-$U_{1/2}$4
A’	H	4.58	4.15	3.84	*3.95*	3.75	*4.17/4.26*			A’-U_2?
β-GlcNAc$_{6S}$	C	103.14	54.37	75.41	*70.88*	77.40	*69.73*			
U_2	H	4.48	3.63	**3.76/3.84**	**3.99**	3.76				
β-D-GlcA	C	106.71	76.71	**79.95/83.82**	**78.77**	79.95	178.21			$U_2$1-$A_1$3
rU	H	3.76/3.81	3.91	**3.94**	**4.06**	4.77				
β-D-GlcA-ol	C	65.01	73.31	**78.61**	**80.25**	75.20	177.23			

[a] Values in boldface and italic font indicate glycosylated and sulfated positions, respectively.
